# Methylthioadenosine-driven rewiring of sulfur assimilation lowers the methyl index and fertility in Arabidopsis

**DOI:** 10.1093/jxb/erag135

**Published:** 2026-03-11

**Authors:** Benjamin J M Tremblay, Saeer A Adeel, Maye Saechao, Yihan Dong, Eric Andrianasolo, John M Steele, Annika Traa, Nilanth Yogadasan, Ishari Waduwara-Jayabahu, Barbara A Katzenback, Rüdiger Hell, Markus Wirtz, Barbara A Moffatt

**Affiliations:** Department of Biology, University of Waterloo, Waterloo, Ontario N2L 3G1, Canada; Department of Biology, University of Waterloo, Waterloo, Ontario N2L 3G1, Canada; Department of Biology, University of Waterloo, Waterloo, Ontario N2L 3G1, Canada; Institut de Biologie Moléculaire des Plantes, UPR2357 du CNRS, Université de Strasbourg, Strasbourg 67084, France; Department of Biology, University of Waterloo, Waterloo, Ontario N2L 3G1, Canada; Department of Biology, University of Waterloo, Waterloo, Ontario N2L 3G1, Canada; Department of Biology, University of Waterloo, Waterloo, Ontario N2L 3G1, Canada; Department of Biology, University of Waterloo, Waterloo, Ontario N2L 3G1, Canada; Department of Biology, University of Waterloo, Waterloo, Ontario N2L 3G1, Canada; Department of Biology, University of Waterloo, Waterloo, Ontario N2L 3G1, Canada; Centre for Organismal Studies (COS), Heidelberg University, D-69120 Heidelberg, Germany; Centre for Organismal Studies (COS), Heidelberg University, D-69120 Heidelberg, Germany; Department of Biology, University of Waterloo, Waterloo, Ontario N2L 3G1, Canada; University of Nebraska-Lincoln, USA

**Keywords:** DNA methylation, methionine salvage, methylthioadenosine, sulfur assimilation, transposable element, Yang cycle

## Abstract

The tight coordination of sulfur metabolism and growth regulation is predicated upon nutrient availability. Central to this balancing act is the utilization of cysteine (Cys) for the formation of methionine (Met) and *S*-adenosylmethionine (SAM). Plants that are severely deficient in regenerating Met due to reduced methylthioadenosine (MTA) nucleosidase activity experience numerous developmental abnormalities. Here, we assess the developmental, metabolic, and regulatory effects associated with decreased MTA recycling. We show that MTA overaccumulation predominantly occurs in reproductive tissues and leads to reduced levels of Cys, Met, and SAM, as well as elevated *S*-adenosylhomocysteine. These disruptions of primary sulfur utilization also lead to the misregulation of energy metabolism and altered cell cycle progression. RNA-seq experiments show a general down-regulation of many developmental and reproductive genes. Targeted metabolite analyses demonstrate clear impacts on the methyl index which are reflected in the results of bisulfite sequencing experiments including global alterations in CG gene body methylation levels and decreases of CHG and CHH methylation in transposable elements. Our findings demonstrate the broad impacts of MTA metabolism on plant development, sulfur utilization, and the maintenance of the methyl index.

## Teaser

Arabidopsis flowers accumulate the sulfur by-product methylthioadenosine when methionine salvage is reduced. This lowers methylation capacity and impairs fertility, but short-term spermidine treatment partly restores growth and reproduction across generations.

## Introduction

Plants continuously monitor their nutrient status and environmental conditions to balance their growth and stress responses. Sulfur utilization is a crucial aspect of this balancing act. Plants fine-tune the flux of available sulfate towards various sulfur-containing compounds to coordinate growth and nutrient limitations, with cysteine (Cys) biosynthesis being at the center of this regulation (Takahashi *et al*., 2011; Watanabe *et al*., 2021). Cys is a source of not only sulfur but also carbon and nitrogen precursors (Takahashi *et al*., 2011). Depending on developmental and environmental cues, Cys utilization determines the flow of sulfur towards *de novo* biosynthesis of glucosinolates (GLSs), glutathione (GSH), or methionine (Met) (Speiser *et al*., 2018).

Met limits the synthesis *S*-adenosylmethionine (SAM), which is the primary methyl donor for diverse methylation reactions, including DNA methylation. A substantial sink for SAM is the synthesis of metabolites including the hormone ethylene (Eth), the polyamines spermine (Spm), spermidine (Spd), and thermospermine (Tspm), and the metal ion chelator nicotianamine (NA; Ravanel *et al*., 1998). Using SAM to synthesize these key metabolites creates methylthioadenosine (MTA) as a by-product (Bürstenbinder *et al*., 2010; Waduwara-Jayabahu *et al*., 2012). The sulfur in MTA is recovered into Met in a group of reactions known as the Yang cycle; the enzyme MTA nucleosidase (MTN) catalyzes the first step in this cycle (Ravanel *et al*., 1998). All *Arabidopsis thaliana* (Arabidopsis) Met salvage pathway mutants except for MTN exhibit no visible phenotypes and have been considered non-essential (Bürstenbinder *et al*., 2007; Friedman *et al*., 2011; Zierer *et al*., 2016).

Earlier analysis of MTN-deficient mutants showed accumulation of MTA to be detrimental in Arabidopsis. MTN activity is encoded by two functionally redundant genes in Arabidopsis: *MTN1* (*AT4G38800*) and *MTN2* (*AT4G34840*) (Bürstenbinder *et al*., 2010). The *mtn1-1mtn2-1* double mutant (with 14% residual MTN activity) accumulates increased levels of MTA even under normal growth conditions, leading to several developmental abnormalities, including delayed bolting, thickened vasculature, as well as both male and female sterility (Waduwara-Jayabahu *et al*., 2012). The basis of these phenotypes remains unknown, though previous studies have suggested that MTA-generating reactions are sensitive to MTA feedback inhibition (Hyodo and Tanaka, 1986; Herbik, 1997; Waduwara-Jayabahu *et al*., 2012). However, of these processes, only NA biosynthesis was decreased in *mtn1-1mtn2-1* plants grown under normal conditions. In fact, MTN-deficient mutants display wild-type-like Eth and Spd content, questioning the hypothesis that MTA feedback inhibition is the dominant reason for the substantial growth defects of *mtn* mutants (Waduwara-Jayabahu *et al*., 2012; Washington *et al*., 2016).

Here, we report our investigations of the biochemical basis for the severe developmental abnormalities associated with MTA accumulation, focusing on the sulfur assimilation pathway and, more specifically, the SAM cycle. Our results show that as MTA accumulates to high levels, a simultaneous reduction in SAM content occurs, indicating the existence of a possible homeostatic mechanism balancing sulfur utilization with MTA accumulation. This decline in sulfur assimilation leads to large-scale transcriptional changes and a loss in methylation potential and DNA hypomethylation, resulting in transposon reactivation.

## Materials and methods

### Plant materials and growth conditions

*Arabidopsis thaliana* (Col-0) seeds were surface-sterilized using chlorine gas and stratified in the dark at 4 °C for 72 h before sowing on half-strength Murashige and Skoog (MS) medium with 0.8% agarose and 1% (w/v) sucrose (Murashige and Skoog, 1962). Seedlings were grown in a tissue culture chamber at 22 °C with continuous 100 µmol m^−2^ s^−1^ photosynthetically active radiation (PAR) for 14 d. Plants to be grown to maturity were transplanted to soil in individual pots and moved to a growth chamber with 150 µmol m^−2^ s^−1^ PAR in long-day conditions. MTA feeding experiments were conducted as described previously (Bürstenbinder *et al*., 2010), sowing seeds on medium with either 500 µM MgSO_4_ or MTA (Abcam: ab141257) in concentrations ranging from 10 µM to 750 µM as a sulfur source and grown for 7 d. Polyamine-supplemented medium was prepared as previously described (Waduwara-Jayabahu *et al*., 2012). Sampling was performed by flash-freezing seedlings or unopened buds in liquid nitrogen before grinding to a powder and storing at −80 °C. The following T-DNA insertion lines were obtained from the Arabidopsis Biological Resource Center (Ohio State University, USA): SALK_085385 (*mtn1-1*), SALK_071127 (*mtn2-1*), and SALK_022510 (*mtn2-5*). The *mtn1-1mtn2-1* and *mtn1-1mtn2-5* lines were generated by crossing as described previously (Waduwara-Jayabahu, 2011; Waduwara-Jayabahu *et al*., 2012). PCR genotyping was performed as described previously (Waduwara-Jayabahu *et al*., 2012) using the primers listed in Supplementary Table S1.

### Plant phenotyping experiments

Vertically grown seedlings were tracked for 14 d by photographing the back of the Petri dishes using a Nikon D3000 camera. The images were then used to measure the primary root length on ImageJ. Root lengths were standardized using an image of a ruler taken at the same focal length. The transition of a plant in soil from vegetative to reproductive development was marked by the visual emergence of the apical floral buds. The bolting time was measured from time of seed sowing to this milestone. At bolting, the number of rosette leaves was counted for each individual plant. Once the plant reached complete senescence, the height of the plant was measured from the base of the primary inflorescence stem to the top, in addition to the total number of siliques and bud clusters. Seeds produced by the plant were carefully harvested throughout its development. To determine the total number of seeds per plant, 500 seeds were initially weighed for each genotype and growth medium. The weight per 500 seeds was then used to quantify the total seed count for each replicate after weighing. The percentage viability of the seeds was subsequently tested on half-strength MS medium.

### Root meristem size measurements

The meristematic zone of seedlings was measured after growth for 7 d on either half-strength MS medium or half-strength MS medium supplemented with 100 µM Spd. Seedlings were harvested from the growth medium, rinsed with 1× phosphate-buffered saline (PBS) on a microscope slide, and the PBS wicked away with a Kimwipe. Samples were then stained with SR2200 cell wall staining solution [SR2200: Renaissance Chemicals; 1% (v/v) DMSO, 0.05% (v/v) Triton X-100, 5% (w/v) glycerol, and 4% (w/v) paraformaldehyde in 1× PBS buffer, pH 8.0] for 5 min at room temperature. Afterwards, staining solution was replaced with 1× PBS and imaged using a Zeiss 700 confocal microscope (Carl Zeiss Inc., Toronto, Canada) with a Plan-Apochromat ×20/0.80 objective with a scan resolution of 1024×1024, 4× scan averaging, and identical gain and laser power across samples. SR2200 was excited with a 405 nm solid-state laser, and emission was recorded between 415 nm and 476 nm. All Zeiss ZEN files were processed using ImageJ. The meristematic zone was defined as the region extending from isodiametric cells of the quiescent center (QC) to the first cell that doubles in length in the cortex cell layer.

### Microscopy of embryonic cellular root morphology

Embryonic root development was assessed by sowing dry seeds on either half-strength MS medium or half-strength MS medium supplemented with 100 µM Spd for 24 h. Mature embryos were dissected out from the seed coat into fixative solution (50% ethanol, 10% acetic acid) for 1 h at room temperature. Embryos were rinsed with 1× PBS, incubated in 1% periodic acid for 40 min, and then rinsed again. Embryos were stained with 100 mg l^–1^ propidium iodide, vacuum infiltrated with staining solution for 2 min at room temperature to improve tissue penetration, and rinsed afterward with 1× PBS. Embryos were then mounted in a chloral hydrate solution (2.7 g of chloral hydrate in 1 ml of water). Stained embryos were imaged using a Zeiss 700 confocal microscope with the same acquisition settings used for seedling imaging and excited with a 488 nm solid-state laser, and emission was recorded between 570 nm and 670 nm. All Zeiss ZEN files were processed using ImageJ. Columella cells as well as QC cells and divisions were counted manually based on point measurements from images taken in the middle Z plane of individual embryos 24 h after imbibition.

### Analysis of callose deposition in anthers

Aniline blue staining was performed to assess callose deposition in anthers by placing whole flowers into 250 µl of fixative solution (1:3 acetic acid:100% ethanol) and vacuum infiltrated for 90 min at room temperature. The fixative solution was exchanged through an ethanol series (70%, 50%, 30%), with each step incubated at room temperature for 10 min, followed by a final rinse with dH_2_O. Flowers were then treated with an 8 M NaOH solution overnight at room temperature. A decolorized, water-soluble aniline blue solution [0.1% (w/v) aniline blue in 108 mM K_3_PO_4_, pH 11] was prepared prior to flower collection and stored at 4 °C for ∼24 h before being filtered through filter paper lined with activated charcoal powder to ensure decolorization. Glycerol was added to a final concentration of 2% (v/v) to provide viscosity and prevent sample drying; the final solution was stored at 4 °C until use. Flowers were then stained in the aniline blue staining solution at room temperature in the dark for 24 h. The flowers were imaged using a Zeiss 700 confocal microscope (Carl Zeiss Inc., Toronto, Canada) with a Plan-Apochromat ×20/0.80 objective, a scan resolution of 1024×1024, 4× scan averaging, and identical gain and laser power across samples. Aniline blue stain was excited with a 405 nm solid-state laser, and emission was recorded between 430 nm and 550 nm. All Zeiss ZEN files were processed using ImageJ.

### HPLC and quantification of metabolites

Metabolites were extracted from ∼100 mg of frozen ground seedling or bud tissue powder using 1 ml of 0.1 M HCl and clarified by centrifugation at 16 000 *g* for 5 min at 4 °C. Extracts were stored at −80 °C. To measure thiols, 200 µl of 1/10th water-diluted extract was first reduced by adding 20 µl of 1 M Tris (pH 8.3), 10 µl of 10 mM DTT, and 25 µl of 0.08 M NaOH before incubating in the dark for 1 h at room temperature. Derivatization was performed by addition of 25 µl of 10 mM monobromobimane in acetonitrile and incubating in the dark for 15 min, before stopping the reaction via addition of 705 µl of 5% acetic acid. Separation was performed using HPLC on a Nova-Pak C18 (4.6 mm×250 mm) column and signal detection with a Jasco 920 Intelligent Fluorescent Detector at 380 nm excitation and 480 nm emission. To measure adenosines, 300 µl of extract was derivatized by addition to 620 µl of 620 mM citric acid-1-hydrate, 760 mM di-sodium hydrogen phosphate di-hydrate (pH 4), and 80 µl of 45% chloroacetaldehyde before incubating at 80 °C for 10 min and subsequently cooling to room temperature on ice. Separation was performed as described earlier, and signal detection used 280 nm emission with 410 nm excitation. For amino acid and polyamine measurements, 5 µl of extract was added to 35 µl of 0.2 M boric acid (pH 8.8) and derivatized by addition of 20 µl of 3 mg ml^−1^ AccQ Taq in acetonitrile and incubating at room temperature for 5 min, followed by incubating at 55 °C for 10 min, and finally diluting 5-fold with water. Separation was performed using HPLC on a Acquity BEH C18 column (150 mm×2.1 mm) in an Acquity H-class UPLC and signal detection with an Acquity FLR-Detector set to 250 nm excitation and 395 nm emission. For NA measurements, 25 µl of extract was added to 75 µl of 0.5 M borate buffer with 5 mM EDTA (pH 7.7) and derivatized by addition to 50 µl of 12 mM 9-fluorenylmethyl chloroformate 9-fluorenylmothoxycarbonyl chloride in acetone before incubating for 45 s at room temperature. The reaction was deactivated by addition of 50 µl of 40 mM adamantan-1-amine amantadine hydrochloride in 75% acetone. Separation was performed using HPLC on a Phenomenex C18 column (4 mm×3 mm) and signal was detected with a Jasco 920 Intelligent Fluorescent Detector at 263 nm excitation and 313 nm emission. Quantification calculations from detection signals were performed using the software Empower Pro. For sulfate measurements, extracts were diluted 10-fold and separated in a Dionex ICS-1000 pump/detector with an Ion-PAC AS 9HC column (2 mm×250 mm). Quantification calculations from detection signals were performed using the software Chromeleon (Dionex). Data analysis was performed using R (version 4.4.1) with packages stats and rstatix (version 0.7.2).

### Mass spectrometry and data analysis

Unopened bud samples were extracted with 1 ml of 0.1 M HCl and clarified by centrifugation at 16 000 *g* for 5 min at 4 °C. Extracts were diluted with deionized water to make 1 mg ml^–1^ samples, then diluted to 1 µg ml^–1^ with 50% acetonitrile with 0.1% formic acid. Using a Velos LTQ Orbitrap Pro (ThermoFisher Scientific), positive and negative electrospray ionization were scanned in an ion trap (low resolution) with scan range 135–1000.

### Target of rapamycin activity assays

For immunological analysis of target of rapamycin (TOR) downstream phosphorylation, total soluble proteins were extracted from 25 mg of frozen unopened buds with a glass bead homogenizer and resuspended in homemade 2× Laemmli buffer at a 1:5 ratio of plant weight (mg) to buffer volume (µl). Samples were vortexed and boiled at 95 °C for 5 min then transferred to ice. Samples were subsequently separated on a 12% 8 M urea SDS–PAGE gel, and were transferred onto a polyvinylidene fluoride membrane. Detection of primary antibodies, S6K-p (Abcam; ab207399; 1:5000) and S6K1/2 (Agrisera; AS12-1855; 1:5000), was accomplished using a horseradish peroxidase-conjugated secondary antibody (1:20 000).

### Flow cytometry

Developmentally matched rosette leaves four and five were harvested from 14-day-old plants that were grown horizontally on half-strength MS medium or half-strength MS medium supplemented with 100 µM Spd, where seeds were sown at a density of 100 seeds per plate. Approximately 20 mg of leaf tissue was finely chopped using a new double-sided razor blade on a pre-chilled watch glass with 600 µl of freshly made ice-cold Aru buffer [Yang et al., 2019; 9.65 ml of MgSO_4_ buffer (10 mM MgSO_4_·7H_2_O, 50 mM KCl, 5 mM HEPES), 1 mM DTT, and 0.1% (v/v) Triton X-100]. The resulting extract was transferred to a 1 ml syringe with a homemade adapter to hold a 40 µm Nitex nylon mesh. The extract was filtered through this adapter into a 5 ml round-bottom polystyrene tube. The suspended nuclei were treated with RNase (50 µg ml^–1^; BioShop, Toronto, Canada) for 10 min on ice and stained with propidium iodide (50 µg ml^–1^). Samples were run on a BD Accuri C6 flow cytometer, operated at a flow rate of 14 µl min^–1^ with acquisition settings as per Galbraith and Lambert (2012). Data analysis was performed using FlowJo software (BD), gating on individual ploidy peaks. The endoreduplication index (EI) was calculated by summing the products of the fraction of events at each ploidy level and the corresponding ploidy level value (i.e. 2C, 4C, 8C, 16C, 32C, or 64C).


$$\mathrm{EI}\;=\frac{4C\;+(2\times 8C)+(3\times 16C)+(4\times 32C)+(5\times \;64C)}{100}.$$

### Methylthioadenosine nucleosidase activity assay

MTN protein activity assays were performed as described by Bürstenbinder *et al*. (2010). Briefly, 20 µg of total protein from unopened bud extracts in 50 mM potassium phosphate buffer (pH 7) were used in a xanthine oxidase-coupled enzyme assay, measuring absorbance at 470 nm every 10 min for 1 h. Enzyme activities were calculated from the rate of change of absorbance.

### RNA-seq experiments and analysis

RNA was extracted from ∼50 mg samples of pooled frozen ground 14-day-old seedling or unopened bud tissue using the RNeasy Mini Kit (Qiagen). RNA was treated with DNase I using the RNase-free DNase Set (Qiagen). Libraries were prepared and sequenced using either a HiSeq2500 or NovaSeq 6000 platform (Illumina) with a PE150 sequencing strategy by Novogene (Beijing, China) or Sickkids (Toronto, Canada). Reads were quality filtered and adapter-trimmed using Trim Galore! (Babraham Bioinformatics) and mapped onto the TAIR10 release of the *A. thaliana* genome (Lamesch *et al*., 2012) with STAR (Dobin *et al*., 2013). Gene-level quantification based on the Araport11 *A. thaliana* genome annotations (Cheng *et al*., 2017) was performed using StringTie (Pertea *et al*., 2015). The edgeR R package was used for differential expression analysis (Robinson *et al*., 2010). Gene Ontology (GO) enrichment analyses were performed for Biological Process (BP) terms using the topGO R package.

### Bisulfite sequencing experiments and analysis

DNA was extracted from ∼50 mg samples of pooled frozen ground unopened bud tissue using the DNeasy Plant Mini Kit (Qiagen). Bisulfite treatment, library preparation, and sequencing using a HiSeq X Ten platform (Illumina) with a PE150 sequencing strategy were performed by Novogene. Reads were quality filtered and adapter-trimmed using Trim Galore! (Babraham Bioinformatics). Read mapping on the TAIR10 release of the *A. thaliana* genome (Lamesch *et al*., 2012), read de-duplication, and cytosine methylation calling were performed using Bismark (Krueger and Andrews, 2011). The DMRcaller R package was used for differential methylation analyses (Catoni *et al*., 2018). Global quantitation plots were created from data obtained using Homer (Heinz *et al*., 2010). GO enrichment analyses were performed for BP terms using the topGO R package.

## Results

### Epigenetically restored *mtn1-1mtn2-1* plants have improved fertility and more normal growth

We previously reported that the fertility of the *mtn1-1mtn2-1* mutant (Fig. 1A) can be partially restored by germinating and growing seedlings for 14 d on medium supplemented with either NA or a polyamine (Spd, Spm, or Tspm) before transferring to soil (Waduwara-Jayabahu *et al*., 2012). Although the basis for this effect was not clear, it provided a means to study the *mtn1-1mtn2-1* phenotype without requiring recovery of the homozygous double mutant from segregating populations. It also offered potential insights into the distinct mechanisms underlying MTA-driven sterility. Initially, we repeated the feeding experiment while carefully monitoring the effects of each compound on seedling growth and fertility. All polyamines restored the reduced primary root length of the double mutant while also leading to a minor increase in viable seed, though this latter effect was only significant for Spd (Fig. 1B, C). We observed that seed obtained from Spd-treated plants generated progeny with greatly improved fertility levels when germinated on Spd-supplemented medium and, by the third generation (G_3_), the plants were substantially more wild-type (WT)-like in appearance, bolting time, and in terms of seed yields compared with untreated *mtn1-1mtn2-1* plants (Fig. 1A, D, E; Supplementary Fig. S1A, B).

**Fig. 1.**
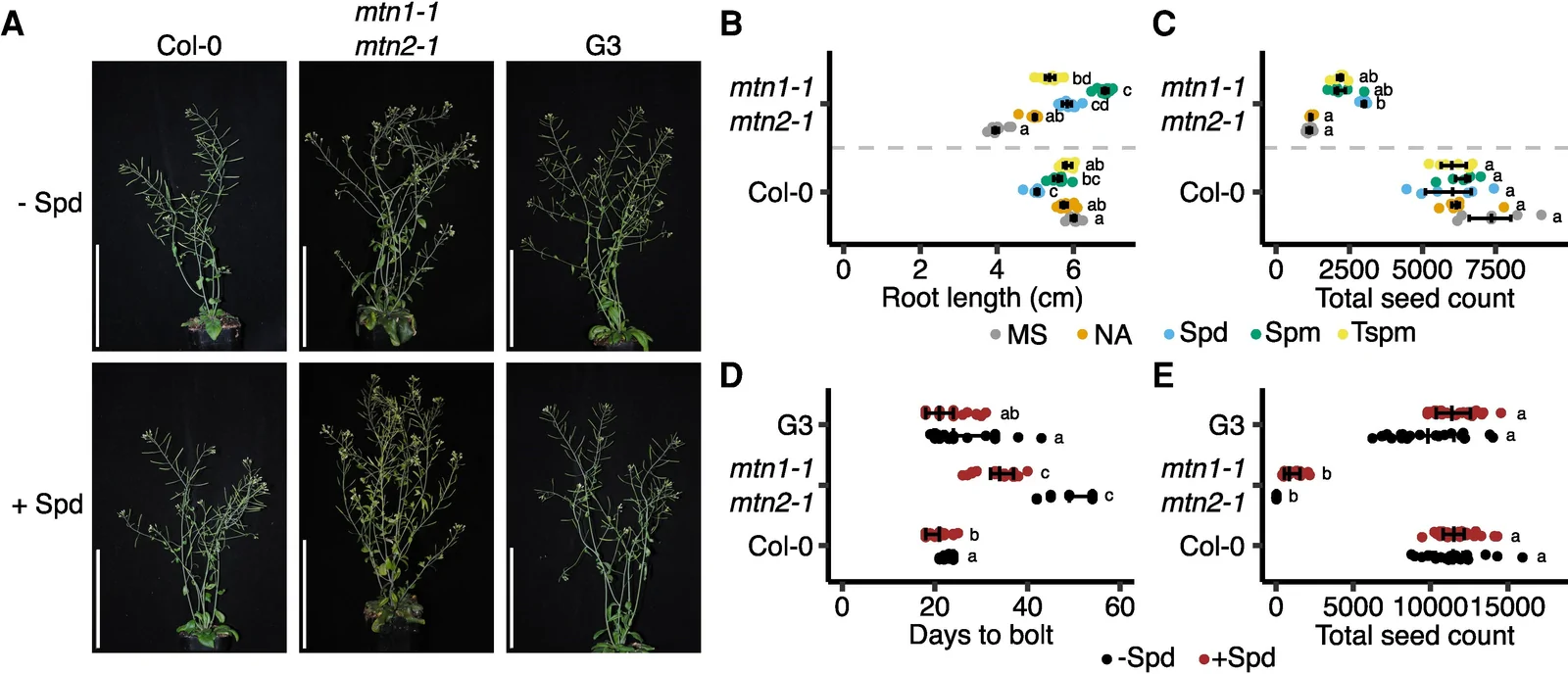
Spd supplementation partially restores *mtn1-1mtn2-1* growth. (A) Col-0 (WT), *mtn1-1mtn2-1*, and G_3_ plants were grown with or without Spd supplementation during the first 2 weeks of growth in Petri plates. These plants were then transplanted into soil and representatives photographed upon silique ripening. Scale bar=10 cm. (B) Primary root lengths of WT and *mtn1-1mtn2-1* seedlings grown in Petri plates for 14 d in the presence of NA, Spd, Spm, or Tspm (*n=*10). Seedlings were also grown in the absence of a treatment (MS). Statistical testing was performed using two-way Kruskal–Wallis tests with Dunn’s post-hoc test. Groups not sharing letters are significantly different (*P*<0.05). (C) Plants exposed to the same treatment regimen as (B) were transplanted to soil and grown to term (*n*=6). Seeds were collected at various stages of maturation and were allowed to dry. The total seed count per plant was assessed by initially weighing 500 seeds for each genotype and growth medium, with the weight per 500 seeds representing the seed yield of each replicate. Seed viability: WT (MS, 100%; NA, 100%; Spd, 100%; Spm, 99%; Tspm, 99%), *mtn1-1mtn2-1* (MS, 98%; NA, 96%; Spd, 100%; Spm, 98%; Tspm, 99%). (D) WT, *mtn1-1mtn2-1*, and G_3_ plants were grown as described for (A), then transplanted to soil where the time till bolting was marked by the visual emergence of the apical floral buds (*n*=17–31). Statistical testing was performed as described for (B). (E) Plants from (D) were grown to term and the total seed count was quantified as described for (C) (*n*=17–31). Seed viability: WT (MS, 100%; Spd, 99%), *mtn1-1mtn2-1* (MS, 98%; Spd, 99%), G_3_ (MS, 100%; Spd, 98%). Statistical testing was performed as described for (B).

Given the broad range of phenotypic consequences of MTN deficiency at various developmental milestones (Waduwara-Jayabahu *et al*., 2012), we sought to assess the extent to which Spd supplementation restored normal development. The marked reduction in primary root length of *mtn1-1mtn2-1* seedlings compared with the WT led us to examine their meristematic development, revealing smaller, and more disordered root apical meristems (Fig. 2A, B). We found that the increase in root length resulting from Spd treatment in *mtn1-1mtn2-1* seedlings (Fig. 1B) coincided with a partial restoration of the meristematic zone, reaching ∼1.5 times the length observed in *mtn1-1mtn2-1* seedlings grown on MS. Notably, the loss of cellular organization within the meristematic zone of the *mtn1-1mtn2-1* mutant grown on MS was partially recovered following Spd treatment. This cellular disorganization was observed not only in the QC but also extended to the columella cells, epidermis, and cortex cell layers. In contrast, QC and columella disorganization was rarely seen in WT or G_3_ seedlings grown on either MS or Spd. G_3_ seedlings exhibited an overall more robust meristematic zone with sizes and organization similar to the WT.

**Fig. 2.**
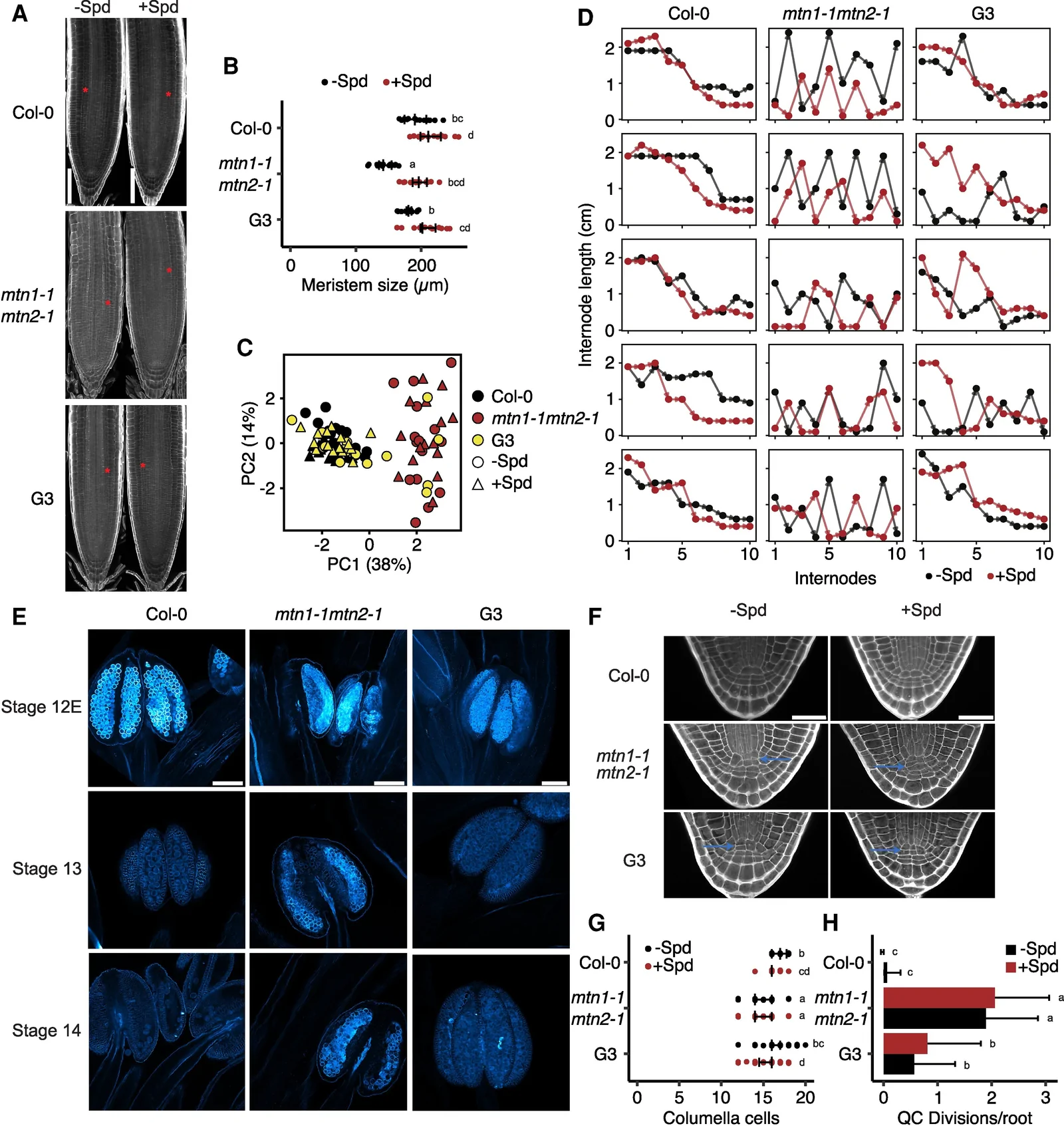
Partial restoration of *mtn1-1mtn2-1* phenotypes across developmental milestones. (A) Representative confocal images of 7-day-old Col-0 (WT), *mtn1-1mtn2-1*, and G_3_ roots obtained using the SR2200 cell wall stain, with the red asterisk indicating the end of the meristematic zone measuring from isodiametric cells of the QC to the first cell that doubles in length in the cortex cell layer. Scale bar=100 µm. (B) The length of the meristematic zone was measured, indicated by the red asterisk in (A). Statistical testing was performed using two-way Kruskal–Wallis tests with Dunn’s post-hoc test. Groups not sharing letters are significantly different (*P*<0.05; *n*=15). (C) After the WT, *mtn1-1mtn2-1*, and G_3_ plants reached ∼25 cm in height, the first 10 internodes between siliques were measured on the primary inflorescence branch for both Spd-treated and untreated plants and summarized in a PCA (*n*=15). (D) The distribution of internode lengths of five representative plants of Spd-treated or untreated WT, *mtn1-1mtn2-1*, and G_3_ based on the internode and its length. (E) Callose deposition in anthers of WT, *mtn1-1mtn2-1*, and G_3_ plants during stages 12E, 13, and 14 of floral development, stained with aniline blue. (F) WT (*n*=38, 47), *mtn1-1mtn2-1* (*n*=53, 37), and G_3_ (*n*=47, 51) seeds were imbibed on medium with or without Spd supplementation for 24 h and the cell walls of the mature embryo root were visualized by propidium iodide staining. Blue arrows indicate QC cells. Scale bar=100 µm. (G) Both the number of columella cells and (H) the number of cell divisions (blue arrow) in the QC were quantified. Statistical testing was performed as in (B).

The improvements in bolting time and seed yield in Spd-treated *mtn1-1mtn2-1* plants prompted us to further investigate the phenotypic effects of Spd on floral development and anther maturation. We measured the lengths of the first 10 internodes of the primary inflorescence branch, and found that the patterns of *mtn1-1mtn2-1* internode lengths were distinct from those of WT and G_3_ plants based on a principal component analysis (PCA), with some variability in the internodes of G_3_ plants germinated without Spd clustering near *mtn1-1mtn2-1* plants (Fig. 2C, D). Overall, *mtn1-1mtn2-1* plants exhibited poorly organized phyllotaxy compared with the WT. The inconsistency of lengths from one internode to another is consistent with previous observations of their shortened plant stature, abnormal plant architecture, and fasciation. The impairment of proper internode development due to MTN deficiency was only partially restored in G_3_ plants, with Spd-treated G_3_ plants showing more consistent internode lengths as seen in the WT. In addition to the abnormal phyllotaxy of the *mtn1-1mtn2-1* mutant, we assessed the level of callose deposition in *mtn1-1mtn2-1* anthers during flower stages 12E to 14, as callose deposition and degradation are fundamental anther development milestones (Sanders *et al*., 1999; Lu *et al*., 2014). Anthers of *mtn1-1mtn2-1* flowers contained clumpy and deformed pollen, and flowers at stages 13 and 14 did not dehisce (i.e. release pollen grains) normally (Fig. 2E). After three generations of growth on Spd-supplemented media, G_3_ plants showed a significant reduction in callose deposition compared with *mtn1-1mtn2-1*, along with the increased release of pollen grains in stage 14 flowers.

Given the severe defects observed in seed production and root apical meristem physiology in *mtn1-1mtn2-1*, we further explored whether MTN deficiency also disrupts embryogenesis in addition to impairing floral meristem development. To evaluate the embryonic development of *mtn1-1mtn2-1* plants, we quantified columella cell numbers and QC divisions of mature *mtn1-1mtn2-1* embryos, as these cell types are established during embryogenesis and provide key developmental insights (Forzani *et al*., 2014). Mutant *mtn1-1mtn2-1* embryos had significantly fewer columella cells than the WT or G_3_ plants grown on media in the absence of Spd supplementation (–Spd), while their QC cells were twice as likely to divide, and exhibit abnormal asymmetrical divisions (Fig. 2F–H). In addition, both *mtn1-1mtn2-1* and G_3_ embryos exhibited irregularities in the cell organization of the root meristem. Although Spd treatment of the mother plants (as seedlings for 14 d) did not induce noticeable effects, G_3_ embryos were nevertheless similar to the WT in terms of their cell organization. Since multiple aspects of plant development are partially restored in G_3_ plants, we saw this transgenerationally stable restoration as an opportunity to examine metabolic and regulatory changes associated with MTA accumulation.

### Moderately methylthioadenosine nucleosidase-deficient *mtn1-1mtn2-5* mutant plants exhibit mild developmental abnormalities and improved fertility

To investigate how varying levels of MTN deficiency impact plant development, we aimed to generate an allelic series of MTN-deficient mutants. By crossing the *mtn1-1* single mutant with a new MTN2 allele, *mtn2-5* (SALK_022510), we generated a moderately MTN-deficient mutant, *mtn1-1mtn2-5*. This double mutant retained 28% residual MTN activity, twice the amount present in the severe *mtn1-1mtn2-1* double mutant (Supplementary Fig. S2A). Phenotypic analysis of *mtn1-1mtn2-5* revealed that its primary root growth was less inhibited by MTA-supplemented medium compared with *mtn1-1mtn2-1*, indicating a clear dose-dependent response to MTA accumulation (Supplementary Fig. S2B). Notably, the additional residual MTN activity in *mtn1-1mtn2-5* substantially mitigated the negative effects of MTA accumulation, restoring much of the primary root growth and significantly reducing delays in bolting observed in the severe mutant (Supplementary Fig. S2B–E). Despite displaying an increased number of bud clusters, indicative of floral meristem activation commonly associated with MTN deficiency, *mtn1-1mtn2-5* remained fertile. Remarkably, it produced more than twice the seed yield of WT plants, contrasting sharply with the sterility of *mtn1-1mtn2-1* (Supplementary Fig. S2F, G). This moderate MTN-deficient mutant demonstrates that even modest changes in MTN activity can significantly affect growth and fertility.

### Increased methylthioadenosine accumulation in buds leads to reduced fertility

As a starting point in our characterization of the metabolic effects of MTA accumulation on development and reproduction, we performed targeted analysis of 29 metabolites, including key compounds related to the SAM cycle and primary sulfur assimilation in seedling and unopened flower bud tissues of our mutants, with and without Spd supplementation (Fig. 3A–C; Supplementary Dataset S1). These measurements revealed that while the *mtn1-1mtn2-1* mutant seedlings accumulate 2-fold higher levels of MTA as compared with the WT, the buds of mature plants contain >10-fold higher levels of MTA (Fig. 3D, E). This led us to hypothesize that reproductive tissues have a greater demand for metabolites whose syntheses generate MTA as a by-product (such as NA and Spd) and thus are more dependent on MTN activity. Steady-state NA levels were lower in buds and seedlings of MTN-deficient mutants (Fig. 3F, G). However, Spd was not affected in any of the MTN mutants (Fig. 3H; Supplementary Fig. S3A). These findings suggest that the biosyntheses of NA and Spd are under differential regulatory control and respond differently to changes in the availability of their precursor compounds. In buds, this may be in part due to increased accumulation of the Spd precursor putrescine (Put) (Supplementary Fig. S3B, C). Alternatively, increases in Put could be a sign of a perceived sulfur deficiency by the plant (Nikiforova *et al*., 2005; Bielecka *et al*., 2015).

**Fig. 3.**
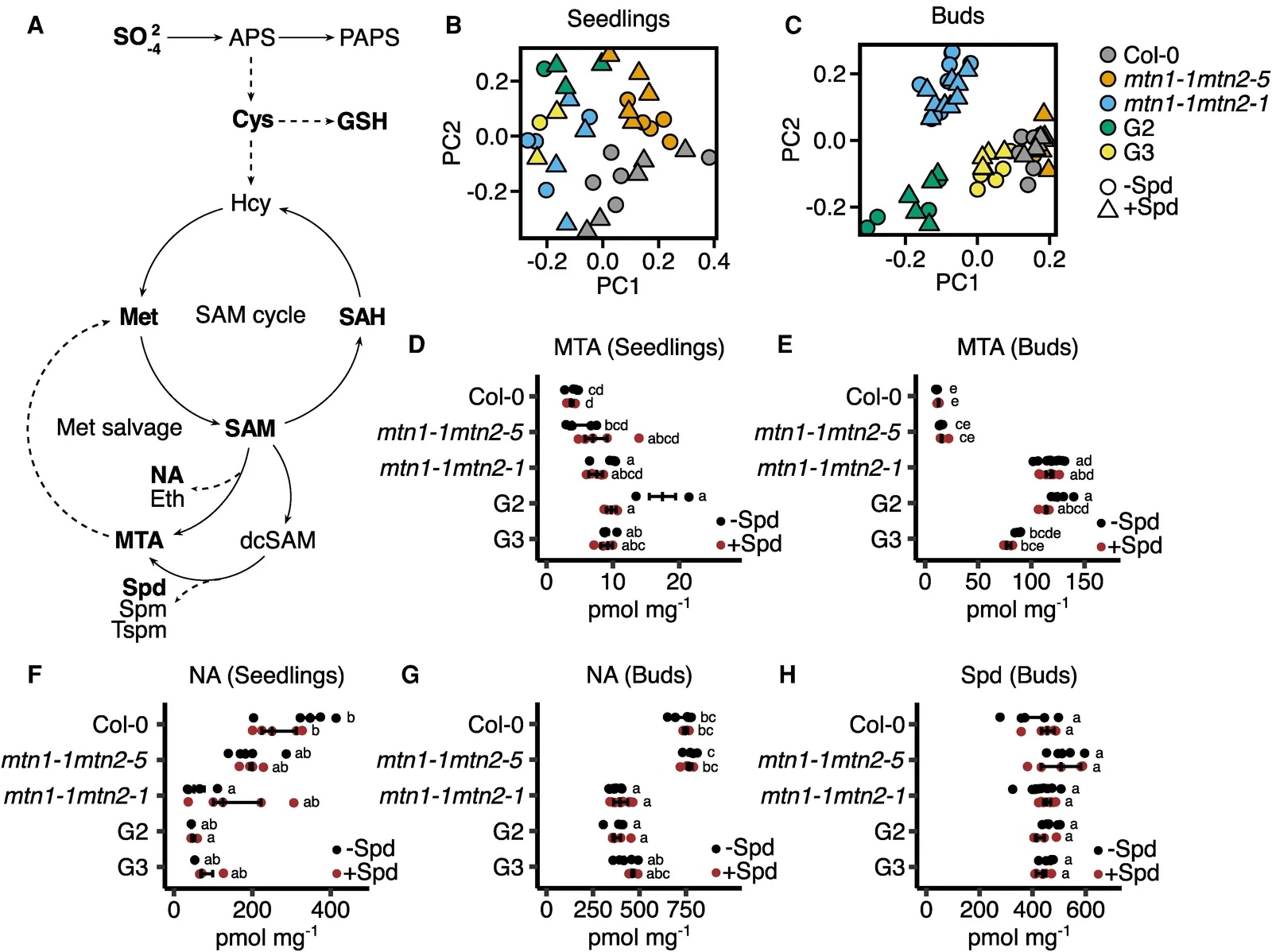
MTA accumulation misregulates primary sulfur assimilation. (A) Schematic of the primary sulfur assimilation pathway, including the SAM cycle and Met salvage. Dashed lines hide intermediate steps. Metabolites in bold have been quantified in this study. (B, C) PCA of all metabolite quantification data from seedlings and buds in this study, respectively, including Col-0 (WT), *mtn1-1mtn2-1*, *mtn1-1mtn2-5*, G_2_, and G_3_ plants. Seedlings were grown in the presence or absence of Spd. (D–H) Individual metabolite quantification data of MTA in seedlings, MTA in buds, NA in seedlings, NA in buds, and Spd in buds, respectively (*n*=4–5). Statistical testing was performed using two-way Kruskal–Wallis tests with Dunn’s post-hoc test. Groups not sharing letters are significantly different (*P*<0.05).

The substantially stronger increase in MTA accumulation in mutant buds compared with seedlings was accompanied by an increased global impact on all measured metabolite levels resulting in greater separation in the PCA between the mutant and WT samples as compared with seedlings (Fig. 3B, C). Interestingly, despite the moderate *mtn1-1mtn2-5* mutants exhibiting developmental abnormalities, the metabolite profile of these plants clustered very close to the WT samples in the PCA of buds. In contrast to *mtn1-1mtn2-1*, the *mtn1-1mtn2-5* mutants did not accumulate significantly higher levels of MTA in buds, probably because the additional residual MTN activity is sufficient to prevent MTA accumulation (Fig. 3D, E). Since the most significant developmental difference between the *mtn1-1mtn2-5* and *mtn1-1mtn2-1* mutants was their fertility, we concluded that the reduced fertility of *mtn1-1mtn2-1* is primarily caused by MTA accumulation in the bud. Indeed, we even observed a slight decrease in MTA accumulation in the more fertile G_3_ buds compared with *mtn1-1mtn2-1* buds.

Additionally, we observed during the process of sampling that removing unopened buds from *mtn1-1mtn2-1* branches often led to subtending flower buds becoming fertile (Supplementary Fig. S4). This observation suggests that individual flowers probably do not accumulate sufficient MTA to cause sterility. Instead, excess MTA accumulated in the bud cluster is likely to be diffused or transported in a basal direction, maintaining the opened flowers in a state of sterility.

### Primary sulfur assimilation metabolites steady-state levels are affected by methylthioadenosine

We next examined the impact of MTA accumulation and MTN deficiency on the SAM cycle (Fig. 3A). Despite being grown in sulfur-sufficient conditions, *mtn1-1mtn2-1* buds contained significantly decreased levels of Met and SAM (Fig. 4A, B). Previous work examining the levels of these metabolites in mutants of other biosynthetic enzymes of the Met salvage pathway revealed that this pathway is only necessary to replenish levels of Met and SAM in sulfur-insufficient conditions, leading us to conclude that MTA itself may be acting in some unknown manner to disrupt regulation of the SAM cycle independent of sulfur availability (Zierer *et al*., 2016). This was further evidenced by elevated levels of *S*-adenosylhomocysteine (SAH) and reduced levels of Cys in the mutant, despite no observable differences in the levels of free sulfate (Fig. 4C–E). Levels of SAM, SAH, and Cys were restored in G_3_ buds, but those of Met were not (Fig. 4A–C, E). In combination with the lack of significant differences for these metabolites in *mtn1-1mtn2-5* buds, these results confirmed the importance of SAM cycle metabolites in maintaining fertility. In particular, SAH accumulation in buds correlated well with MTA accumulation (Pearson *r*=0.913; Supplementary Table S2) and decreased fertility.

**Fig. 4.**
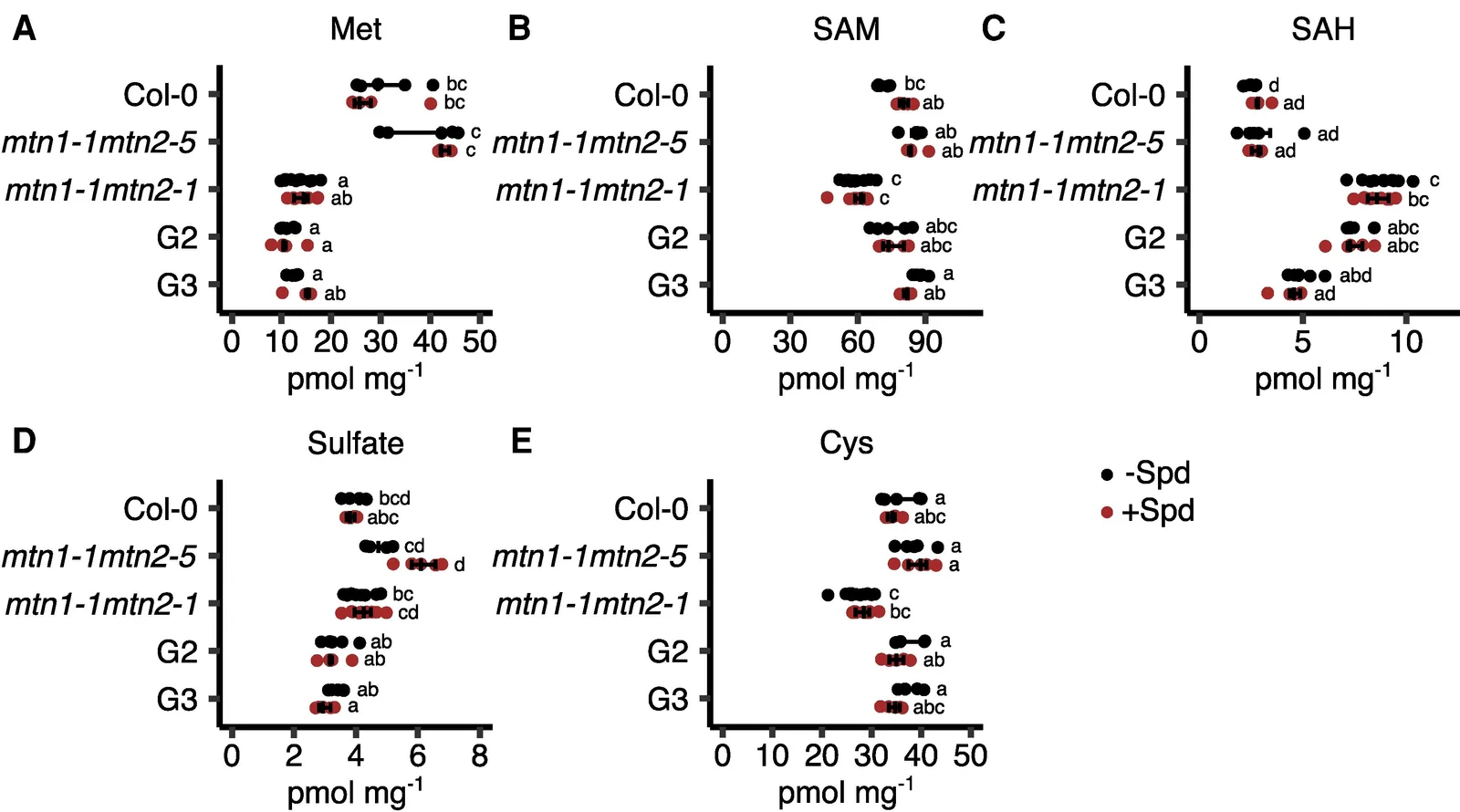
Effects of MTA accumulation on SAM cycle metabolites is independent of sulfate levels. (A–E) Individual metabolite quantification data of Met, SAM, SAH, sulfate, and Cys, respectively, from buds of Col-0 (WT), *mtn1-1mtn2-1*, *mtn1-1mtn2-5*, G_2_, and G_3_ plants having been grown in the presence or absence of Spd supplementation (*n=*4–5). Statistical testing was performed using two-way Kruskal–Wallis tests with Dunn’s post-hoc test. Groups not sharing letters are significantly different (*P*<0.05).

### Altered energy metabolism and cell cycle changes are probably secondary effects of methylthioadenosine

Since the Yang cycle recycles the sulfur moiety in MTA, we investigated the effects of the MTN-deficient phenotype on sulfur-sensing mechanisms in plants. Previous work has shown that plants sense sulfur deficiency and reduce their growth via the TOR pathway, which acts as a master regulator of energy metabolism (Dong *et al*., 2022). We first examined the levels of the amino acids which we had measured in our metabolite measurement experiments, as regulation of TOR function requires proper homeostasis of free amino acid levels (Kim and Guan, 2019). There were slight increases in the abundances of most of the measured amino acids in *mtn1-1mtn2-1* seedlings, which were generally unchanged in G_3_ seedlings (Fig. 5A). In buds, there was a wider range of both increased and decreased levels of amino acids in *mtn1-1mtn2-1* plants. Reductions of the levels of Ile, Leu, Lys, and Tyr were reversed in G_3_ buds, indicating MTA-dependent misregulation of amino acid metabolism in *mtn1-1mtn2-1* plants.

**Fig. 5.**
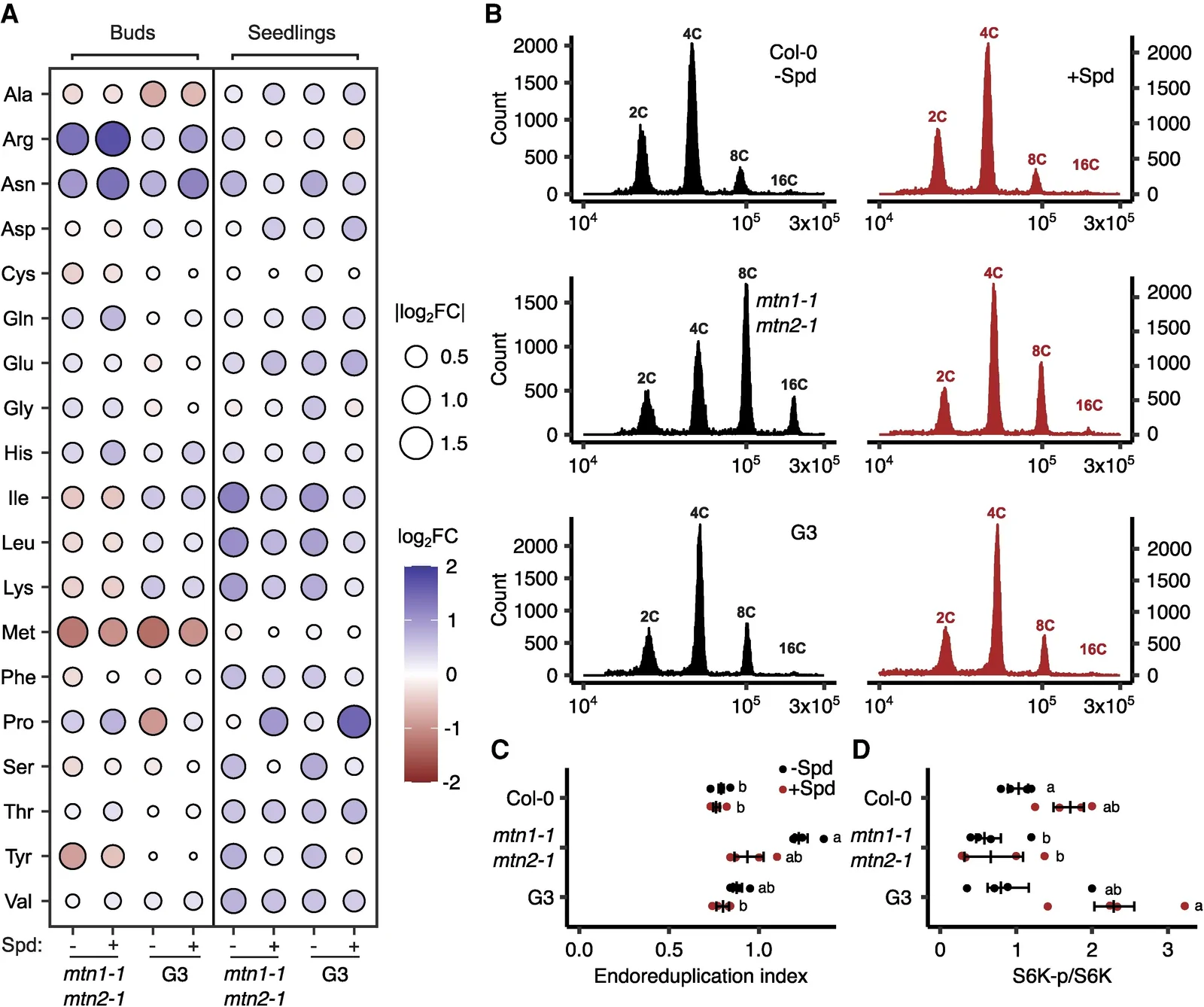
Lack of clear evidence of TOR misregulation by MTA. (A) Average fold change (FC) levels of 19 amino acids in *mtn1-1mtn2-1* and G_3_ seedlings and buds, with or without Spd supplementation, compared with Col-0 (WT; *n*=4–5). The log_2_ FC levels are shown by color, and the absolute magnitude is shown by the size of the circle. (B) The cell ploidy of 14-day-old WT, *mtn1-1mtn2-1*, and G_3_ seedlings grown horizontally on MS (black-filled histogram plots) and Spd (red-filled histogram plots) media (*n*=4) were measured using flow cytometry with developmentally matched rosette leaves. Four major peaks in these representative histograms are from cells with ploidy levels of 2C, 4C, 8C, and 16C, respectively. (C) The endoreduplication index was calculated from the percentage of isolated nuclei within each major peak. Statistical testing was performed using two-way Kruskal–Wallis tests with Dunn’s post-hoc test. Groups not sharing letters are significantly different (*P*<0.05; *n*=4). (D) Ratio of phosphorylated S6K (S6K-p) to unphosphorylated S6K (as a measure of TOR activity) in WT, *mtn1-1mtn2-1*, and G_3_ buds grown with or without Spd supplementation (*n*=4). Statistical testing was performed as for (C).

We next investigated whether MTA accumulation in *mtn1-1mtn2-1* plants led to an impairment in the cell cycle and meristem activity, which are under tight regulation of the TOR signaling network (Pfeiffer *et al*., 2016). We observed early induction of the endocycle in *mtn1-1mtn2-1* mutants (Fig. 5B, C). These plants had an endoreduplication index ∼1.5-fold higher than WT plants, alongside a substantial pool of 8C and 16C nuclei, which was largely recovered in G_3_ plants. Finally, we measured levels of the phosphorylated and unphosphorylated forms of S6K, the downstream target of TOR kinase activity (Wullschleger *et al*., 2006). Although we could detect reduced average levels of S6K (both phosphorylated and unphosphorylated) in *mtn1-1mtn2-1* buds, as well as a reduced ratio of phosphorylated to unphosphorylated S6K, these changes were not significantly different from the WT (Fig. 5D; Supplementary Fig. S5). The lack of clear evidence for disruption of TOR activity in a manner linked to MTA accumulation led us to conclude that while MTN deficiency is likely to be associated with alterations in energy metabolism (via changes in free amino acid levels) and cell cycle progression, these changes may be secondary to other effects of MTA accumulation.

### Differential expression of developmental genes in *mtn1-1mtn2-1* buds

To provide further evidence for our hypothesis that MTA accumulation is a main driver of the observed developmental differences in MTN-deficient mutants, we next compared their transcriptomes. Since *mtn1-1mtn2-1* and *mtn1-1mtn2-5* seedlings did not accumulate much more MTA, we expected to find only minor changes in their transcriptomes. Indeed, the transcriptomes of the mutant and WT seedlings were highly correlated, with few differentially expressed genes between *mtn1-1mtn2-1* and the WT (Fig. 6A; Supplementary Fig. S6; Supplementary Dataset S2). We then proceeded with an RNA-seq experiment using buds from *mtn1-1mtn2-1* mutants, which do accumulate much higher levels of MTA, as well as buds from the partially recovered G_3_ plants. This time we observed larger differences in the overall transcriptome of *mtn1-1mtn2-1* mutants as compared with the WT (Fig. 6B; Supplementary Fig. S7; Supplementary Dataset S3). The majority of differentially expressed genes in *mtn1-1mtn2-1* buds were strongly down-regulated. A GO enrichment analysis revealed that many of these genes were involved in cell wall organization, extracellular region, and pollination, among others (Fig. 6C; Supplementary Dataset S4). None of the enriched GO terms was directly related to Eth, supporting previous evidence that Eth levels are not affected in *mtn1-1mtn2-1* plants (Washington *et al*., 2016). Spd treatment was specifically further associated in the differential expression of genes regulating the response to abiotic stimulus and hypoxia. A large proportion of the differentially expressed genes in *mtn1-1mtn2-1* buds had the opposite differential expression pattern when comparing G_3_ and *mtn1-1mtn2-1* buds, suggesting an association between these specific pathways and the lack of fertility of the mutant (Fig. 6D, E).

**Fig. 6.**
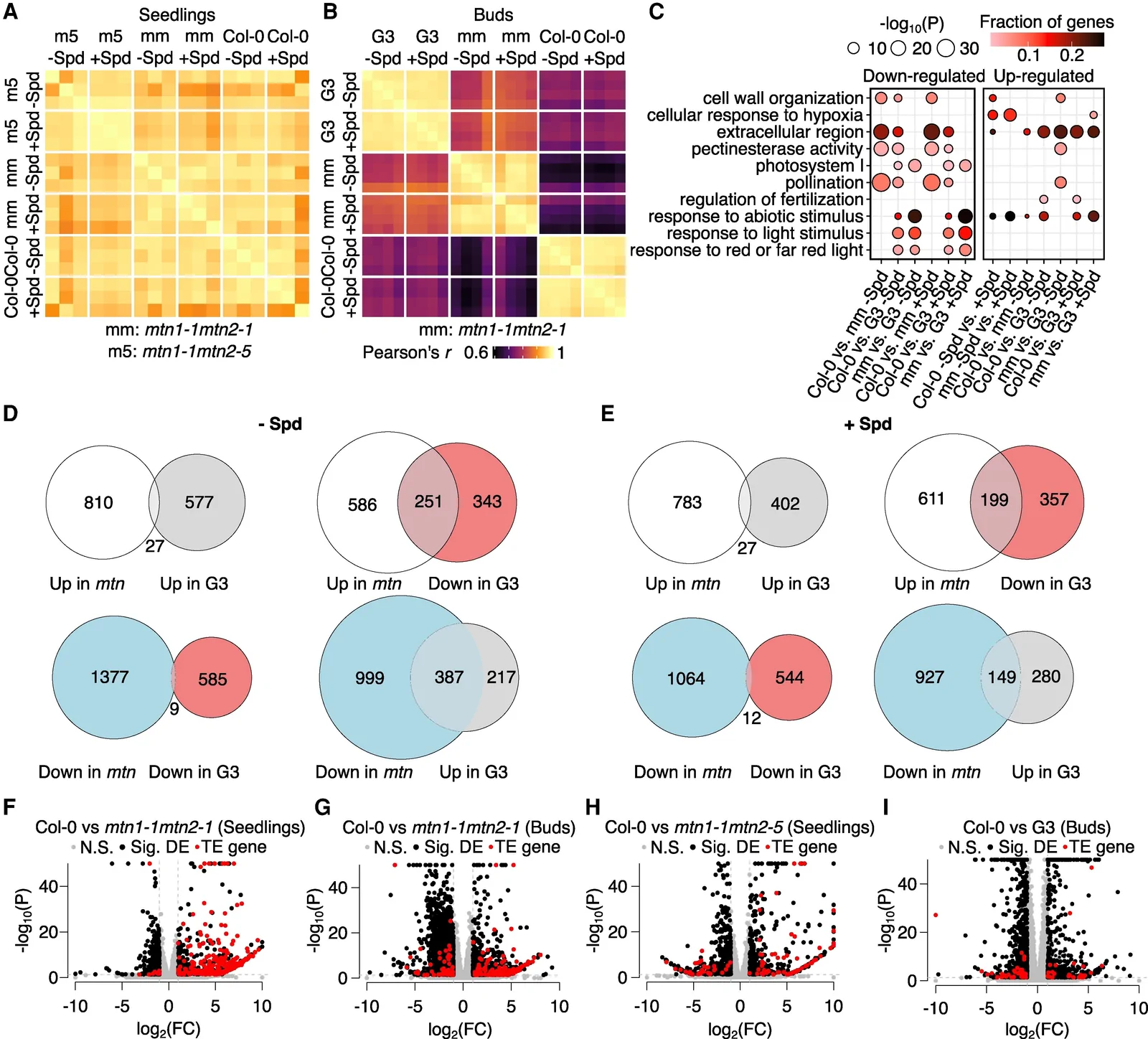
Major transcriptional changes underpin the *mtn1-1mtn2-1* phenotype. (A) Pearson correlation coefficient (*r*) matrix from RNA-seq transcript quantification of Col-0 (WT), *mtn1-1mtn2-1* (mm), and *mtn1-1mtn2-5* (m5) seedlings with or without Spd supplementation. (B) Same as (A) for RNA-seq transcript quantification of WT, *mtn1-1mtn2-1* (mm), and G_3_ buds grown with or without Spd supplementation. (C) Enriched GO terms from up- and down-regulated gene sets between WT, *mtn1-1mtn2-1* (mm), and G_3_ comparisons of differentially expressed genes. The size of the circle represents the magnitude of the −log_10_ of the *P*-value, and the color represents the fraction of genes in each gene set associated with the enriched GO term. (D and E) Venn diagrams of the number of overlapping differentially expressed genes between *mtn1-1mtn2-1* (*mtn*) plants versus WT and G_3_ plants versus *mtn1-1mtn2-1*, with or without Spd supplementation. (F–I) Volcano plots of the −log_10_  *P*-value and log_2_ fold change (FC) of differentially expressed genes between WT, *mtn1-1mtn2-1*, and *mtn1-1mtn2-5* comparisons in seedling and bud RNA-seq. Points representing significantly differentially expressed genes are colored in black (as opposed to gray for non-significant), and significantly differentially expressed transposable element genes are colored in red.

### Abnormal distribution of CG methylation and reduced levels of CHG and CHH methylation lead to gene silencing and transposable element hypomethylation

The differential gene expression analysis also revealed that many genes annotated as ‘transposable_element_gene’ in Araport11 (Cheng *et al*., 2017) were up-regulated in *mtn1-1mtn2-1* buds (Fig. 6F–I). We hypothesized that the up-regulation of transposable element genes could be due to their hypomethylation, as they are normally silenced by DNA methylation. A loss of methylation potential was supported by a decreased SAM/SAH ratio in buds, also known as the methyl index, an indicator of methylation capacity (Fig. 7A, B) (Hoffman *et al*., 1980; Caudill *et al*., 2001). We proceeded with profiling genome-wide DNA methylation in buds using bisulfite sequencing (BS-seq) which, in addition to profiling the methylation status of transposable elements, would allow us to determine whether any potentially heritable DNA methylation changes were associated with the epigenetic fertility restoration phenotype of G_3_ plants.

**Fig. 7.**
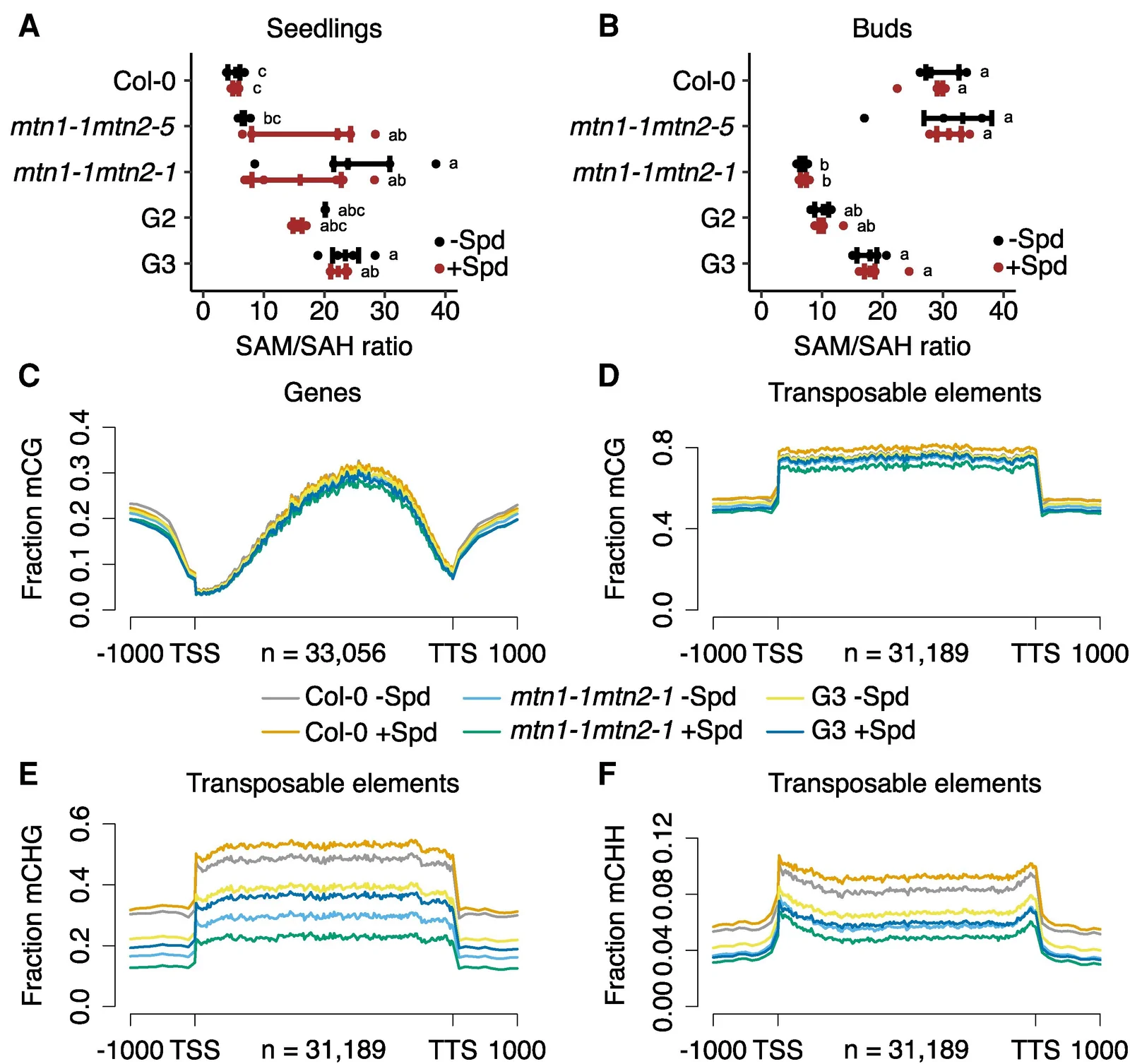
MTA accumulation is associated with major loss of CHG DNA methylation. (A, B) Ratio of SAM to SAH in seedlings and buds, respectively, as an indicator of the methyl index. Quantification data are from Col-0 (WT), *mtn1-1mtn2-1*, *mtn1-1mtn2-5*, G_2_, and G_3_ plants with or without Spd supplementation (*n*=4–5). Statistical testing was performed using two-way Kruskal–Wallis tests with Dunn’s post-hoc test. Groups not sharing letters are significantly different (*P*<0.05). (C, D) Metagene plots of average CG DNA methylation, including 1 kbp upstream and downstream, for genes and transposable elements, respectively, from the Araport11 annotations. DNA methylation data are from BS-seq of WT, *mtn1-1mtn2-1*, and G_3_ buds, with or without Spd supplementation. (E, F) Metagene plots of average CHG and CHH DNA methylation, respectively, of transposable elements as for (D).

Our analysis revealed thousands of differentially methylated regions (DMRs) in the CG context in *mtn1-1mtn2-1* and G_3_ buds compared with the WT, in terms of both hypo- and hypermethylation (Supplementary Fig. S8; Supplementary Dataset S5). Many of these CG DMRs were in common between *mtn1-1mtn2-1* and G_3_ buds; however, we observed that 462 hypermethylated DMRs in *mtn1-1mtn2-1* buds were in turn hypomethylated in G_3_ buds. The presence of a large number of DMRs did not affect the average profile of methylated CGs over gene bodies or transposable elements, suggesting that these events are probably not due to systematic changes in CG methylation capacity (Fig. 7C, D). Indeed, many of these CG DMRs were specifically enriched in down-regulated genes in the RNA-seq related to cell and pollen growth GO terms (Supplementary Fig. S9). Spd appeared to have little effect, with no detectable changes in WT methylation and relatively few DMRs in *mtn1-1mtn2-1* and G_3_ buds (Supplementary Fig. S10).

MTA accumulation appeared to instead have a much greater effect on global methylation levels in the CHG and CHH contexts. We observed a near 50% decrease in average CHG and CHH methylation in transposable elements in *mtn1-1mtn2-1* buds, with partial recovery in G_3_ buds (Fig. 7E, F). There were 4957 hypomethylated DMRs in the CHG context in *mtn1-1mtn2-1* buds, compared with only five hypermethylated DMRs (Supplementary Fig. S8). G_3_ buds had far fewer CHG context DMRs, with 887 hypomethylated (of which 457 were in common with *mtn1-1mtn2-1*) and 11 hypermethylated DMRs. As before Spd appeared to have little effect within the same generation (Supplementary Fig. S10). Far fewer CHH context DMRs could be identified, probably as a result of methylation in this context being present in low amounts (Supplementary Fig. S8C).

### Possible detection of increased secondary sulfur assimilation in *mtn1-1mtn2-1*

In the course of our HPLC measurements of SAM, SAH, and MTA in WT and *mtn1-1mtn2-1* buds, we noticed additional peaks in the resulting chromatograms, with significantly higher signal in the mutant sample (Supplementary Fig. S11A). We suspected that these peaks could be indicative of increased levels of adenosine-containing compounds such as adenosine 5-phosphosulfate (APS), 3-phosphoadenosine 5-phosphosulfate (PAPS), or 3-phosphoadenosine 5-phosphate (PAP) as a result of increased secondary sulfur assimilation (Fig. 3A), though we lacked the necessary standards to test this theory. As an alternative, we performed LC/MS to qualitatively compare any WT and *mtn1-1mtn2-1* LC/MS peaks with expected mass (*m/z*) and retention patterns indicative of these compounds. Using ion trap negative mode, we detected a peak in the *mtn1-1mtn2-1* sample with a mass of 507.9234 *m/z*, absent from the WT, possibly matching PAPS (Supplementary Fig. S11B). With ion trap positive mode, we detected peaks with mass 287.0874 *m/z* and 355.1719 *m/z* in *mtn1-1mtn2-1*, absent in the WT, matching the expected mass of adenosine and APS, respectively (Supplementary Fig. S11C). Two additional peaks with mass 433.1733 *m/z* and 595.2594 *m/z*, matching the expected values of PAP and PAPS, respectively, could be detected in both samples. From these results, we hypothesized that normal levels of sulfate in *mtn1-1mtn2-1*, in combination with reduced primary sulfur assimilation, is leading to increased APS and PAPS, indicative of increased secondary sulfur assimilation. Additional work will be required to validate these data.

## Discussion

The Yang cycle plays an important role in allowing plants to replenish their pools of SAM without requiring additional sulfur inputs (Fig. 3A) (Bürstenbinder *et al*., 2010; Zierer *et al*., 2016). SAM is an essential compound which is used as a methyl donor in many methylation reactions throughout cells and is a precursor for the biosynthesis of Eth, NA, and polyamines. While loss of a functional Yang cycle does not impair development of Arabidopsis under normal conditions, near total loss of MTN activity associated with overaccumulation of MTA leads to several developmental and reproductive deficiencies even under sulfur-sufficient conditions (Waduwara-Jayabahu *et al*., 2012; Zierer *et al*., 2016). Making use of moderately deficient MTN mutants confirmed the reproductive failure phenotype seen in severe *mtn1-1mtn2-1* mutants to be specific to their high levels of MTA, with a tipping point for sterility between 14% and 28% residual MTN activity. Mutant *mtn1-1mtn2-1* second- and third-generation transgenerationally restored plants (G_2_ and G_3_, respectively) by treatment of seedlings for 14 d with Spd also had decreased levels of MTA in buds in association with their increase in fertility and partial restoration of other phenotypic abnormalities (Figs 1–3). As the effects of the treatment persist into the reproductive stage and even to subsequent generations, Spd is likely to be inducing heritable epigenetic changes via chromatin modifications or DNA methylation, though we could not find clear evidence of the latter from our BS-seq data. One possible reason for this is that fertility-linked changes are only present within a subset of cells, which are missed by our bulk tissue sampling. Overall, the data establish a clear link between high levels of MTA accumulation, misregulation of primary sulfur metabolism, and reproductive failure irrespective of the actual sulfur levels available to the plant.

To further explore the link between changes in primary sulfur metabolism and phenotypic effects, we performed RNA-seq of MTN-deficient seedlings and buds. The high levels of MTA accumulation in buds of MTN-deficient mutants were linked to large numbers of differentially expressed genes, including those in pathways related to fertilization. Additionally, we noticed an up-regulation in the expression of transposable element genes in both *mtn1-1mtn2-1* seedlings and buds (Fig. 6F–I). Increased expression of transposable element genes has been linked to stress responses and global DNA hypomethylation in plants (Hudson *et al*., 2011; Roquis *et al*., 2021). Our RNA-seq experiment showed clear signs of up-regulation of stress-related genes in *mtn1-1mtn2-1* plants. Furthermore, a previous report has demonstrated DNA hypomethylation in *hog1* mutant plants which lack the primary SAH hydrolase enzyme activity, leading to a disruption in the SAM cycle and reduced fertility (Ouyang *et al*., 2012). The reduction in DNA methylation in *hog1* mutants is likely to be the result of the decrease in SAM (or an increase in SAH leading to feedback inhibition of methylation reactions), similar to what we observed in *mtn1-1mtn2-1* mutants (Fig. 4A–C). An opposite but related phenomenon has been observed in plants with increased cystathionine γ-synthase expression, which leads to increased Met biosynthesis and increased DNA methylation (Girija *et al*., 2023). BS-seq of *mtn1-1mtn2-1* buds revealed a near 50% decrease in CHG and CHH methylation at sites which are involved in small RNA-guided silencing of transposable element methylation (Fig. 7E, F) (Van Ex *et al*., 2011). Buds of G_3_ plants, which have reduced levels of MTA, had intermediate levels of mCHG and mCHH over transposable elements. This suggests that the methylation potential of the plant is highly sensitive to varying levels of MTA, with its accumulation having a direct impact on the availability of SAM for methylation, which in plants is tightly linked with phenotype such as via epialleles which regulate the expression of nearby genes (Baduel *et al*., 2025).

Plants finely regulate sulfur metabolism to balance growth and stress responses, with Cys biosynthesis playing a central role in this coordination. Our study reveals that MTA accumulation disrupts this delicate balance, causing severe misregulation of primary sulfur utilization, resulting in reduced levels of Cys, Met, and SAM, and impairing downstream processes such as DNA methylation and NA biosynthesis. The reduced utilization of sulfur for Cys biosynthesis suggests a shift in sulfur assimilation toward secondary metabolism, such as GLS biosynthesis, in MTN-deficient mutants. This is further supported by our detection of additional, highly abundant unknown compounds in *mtn1-1mtn2-1* samples from HPLC experiments, potentially linked to elevated secondary sulfur metabolites used for GLS biosynthesis (Supplementary Fig. S11). While the majority of GLSs are converted into simple nitriles, a recent study suggested that sulfur is reallocated back to Cys production in a retrograde pathway under sulfur deprivation (Sugiyama *et al*., 2021). MTN-deficient plants may be utilizing this avenue for relieving the deprivation of primary sulfur metabolites.

Beyond its impact on sulfur assimilation, MTA accumulation influences various developmental pathways, underscoring its wide-reaching effects on plant growth, as evidenced by altered cell cycle progression, root meristems, and impaired floral development (Figs 2, 5). These effects are probably due to interactions between MTA and a set of unknown proteins or metabolites. Whether the phenotypic consequences are direct or secondary downstream effects is currently unknown. As we have shown previously, a lack of MTN protein is itself not harmful to the plant, only the lack of its enzymatic activity (Waduwara-Jayabahu *et al*., 2012). The ability of a plant to overcome the disruptions caused by MTA accumulation appears to play a critical role in determining its fertility, as seen in G_3_ mutants, where slightly lower MTA levels than in *mtn1-1mtn2-1* mutants correlate with partially restored fertility. In conclusion, these data demonstrate the essential role of MTN activity in plant sulfur utilization, independent of other enzymes in the Yang cycle, highlighting its critical contribution to growth and development.

## Supplementary Material

erag135_Supplementary_Data

## Data Availability

All RNA-seq and BS-seq datasets generated in this study are available from the NCBI GEO repository under the accession GSE288809 (https://www.ncbi.nlm.nih.gov/geo/query/acc.cgi?acc=GSE288809). Raw data for phenotypic measurements can be found on Zenodo under the DOI 10.5281/zenodo.18282656 (https://zenodo.org/records/18282656). HPLC data can be found in Supplementary Dataset S1. The remaining primary data supporting this study (confocal microscopy images and flow cytometry data) were not made publicly available at the time of publication. Data will be made available upon request to the corresponding author.

## Supplementary data

The following supplementary data are available at *JXB* online.

Fig. S1. Further phenotypic analyses of the effect of Spd on *mtn1-1mtn2-1* growth.

Fig. S2. Analysis of the intermediate *mtn1-1mtn2-5* mutant.

Fig. S3. Polyamine levels in MTN-deficient plants.

Fig. S4. Fertility restoration by unopened bud decapitation in *mtn1-1mtn2-1*.

Fig. S5. Additional data on S6K phosphorylation levels in *mtn1-1mtn2-1*.

Fig. S6. Transcriptome changes in *mtn1-1mtn2-1* seedlings.

Fig. S7. Transcriptome changes in *mtn1-1mtn2-1* buds.

Fig. S8. Global changes in DNA methylation in *mtn1-1mtn2-1* and G_3_ plants.

Fig. S9. Association between changes in DNA methylation in *mtn1-1mtn2-1* and changes in transcript abundance of many pollen genes.

Fig. S10. The effect of within-generation Spd supplementation on DNA methylation.

Fig. S11. Unknown peaks in HPLC data potentially indicative of increased APS and PAPS in *mtn1-1mtn2-1*.

Table S1. Genotyping primers used in this study.

Table S2. Correlations between MTA and other measured metabolites from buds.

Dataset S1. Seedling and bud metabolite quantification.

Dataset S2. Seedling RNA-seq fold changes.

Dataset S3. Bud RNA-seq fold changes.

Dataset S4. Bud RNA-seq Gene Ontology enrichment.

Dataset S5. Bud BS-seq differentially methylated regions.

Dataset S6. *P*-values associated with significant difference testing.
