# Supplementary material for: Methylthioadenosine-driven rewiring of sulfur assimilation lowers the methyl index and fertility in Arabidopsis
Source: J Exp Bot. 2026 Mar 11;77(18):6209–23. doi: 10.1093/jxb/erag135 (PMC13621326; doi:10.1093/jxb/erag135)
Supplement: Supplementary file 1 [file erag135_supplementary_data.zip › jexbot317052-file001.pdf]

SUPPLEMENTARY: Methylthioadenosine-driven rewiring of sulfur assimilation lowers the methyl index and fertility in Arabidopsis

Benjamin J.M. Tremblay[1*], Saeer A. Adeel[1], Maye Saechao[1], Yihan Dong[2], Eric Andrianasolo[1], John M. Steele[1], Annika Traa[1], Nilanth Yogadasan[1], Ishari Waduwara-Jayabahu[1], Barbara A. Katzenback[1], Rüdiger Hell[3], Markus Wirtz[3], Barbara A. Moffatt[1]

[1] Department of Biology, University of Waterloo, Waterloo, Ontario, Canada N2L 3G1
[2] Institut de Biologie Moléculaire des Plantes, UPR2357 du CNRS, Université de Strasbourg, Strasbourg, 67084, France
[3] Centre for Organismal Studies (COS), Heidelberg University, 69120 Heidelberg, Germany

*To whom correspondence should be addressed.
Contact: benjamin.tremblay@uwaterloo.ca

Supplementary Figures **p. 2-14**

Supplementary Tables **p. 15-16**

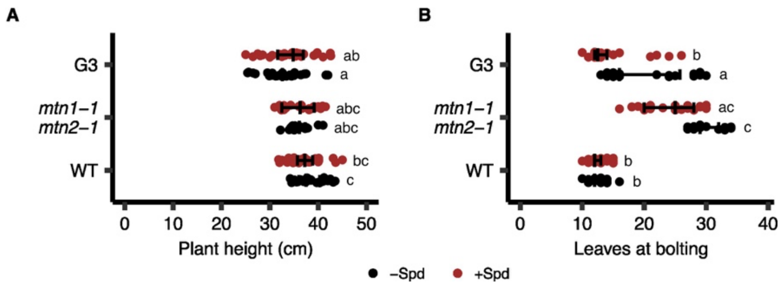

Supplementary Figure S1: Further phenotypic analyses of the effect of Spd on *mtn1-1mtn2-1* growth.

A Col-0, *mtn1-1mtn2-1*, and G3 plants were grown with or without spermidine (Spd) supplementation during the first two weeks of growth in petri plates (n = 17-31), and then transplanted into soil. The plant height was measured from the base of the primary inflorescence stem to the top when the apical bud cluster failed to produce new flowers. Statistical testing was performed using two-way Kruskal-Wallis tests with Dunn's post-hoc test. Groups not sharing letters are significantly different ($P < 0.05$). **B** The transition from vegetative growth to flowering was marked by the emergence of the floral bud cluster, at which point the rosette leaves were counted. Statistical testing was performed as per **A**.

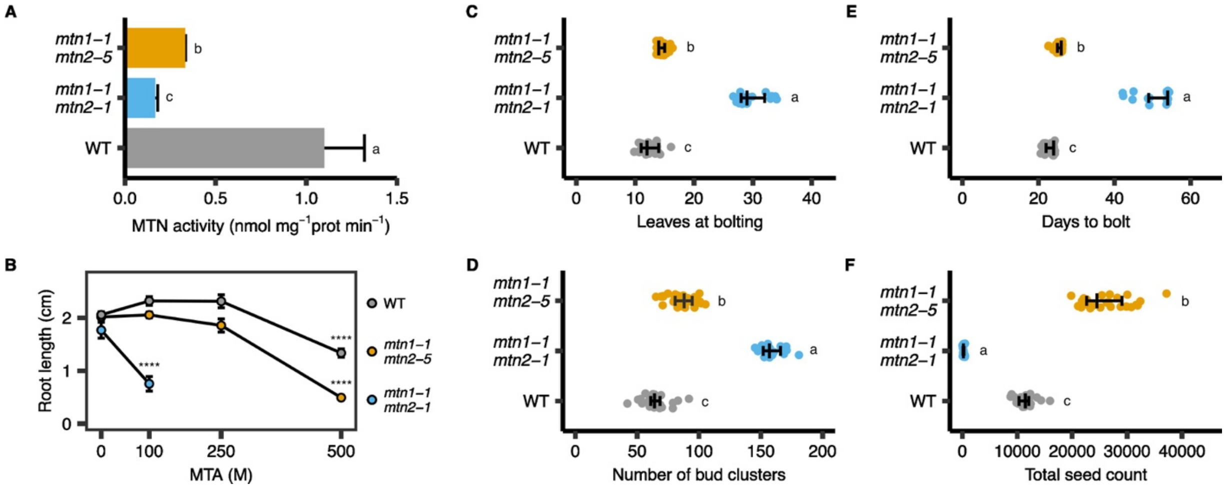

Supplementary Figure S2: The *mtn1-1mtn2-5* mutant displays an intermediate phenotype.

A MTN activity assays performed using bud clusters from WT, *mtn1-1mtn2-1*, and *mtn1-1mtn2-5* plants (n = 3). Data from WT and *mtn1-1mtn2-1* plants have been previously published (Waduwara-Jayabahu et al., 2012). All samples are from the same experiment. Statistical testing was performed using two-sided Student's t-tests with Holm correction for multiple testing. Groups not sharing letters are significantly different (P < 0.05). **B** Primary root lengths of Col-0 (WT), *mtn1-1mtn2-1*, and *mtn1-1mtn2-5* plants grown on plates containing MTA-supplemented medium (n = 8-64). Statistical testing was performed using two-way Kruskal-Wallis tests with Dunn's post-hoc test. Only comparisons to Col-0 are shown (****P<0.0001). **C-F** Phenotyping data of Col-0 (WT), *mtn1-1mtn2-1*, and *mtn1-1mtn2-5* plants (n = 17-32), including the number of leaves at bolting, number of days until bolting, the number of bud clusters at senescence, and total seed count upon harvest, respectively. Statistical testing was performed using two-way Kruskal-Wallis tests with Dunn's post-hoc test. Groups not sharing letters are significantly different (P < 0.05).

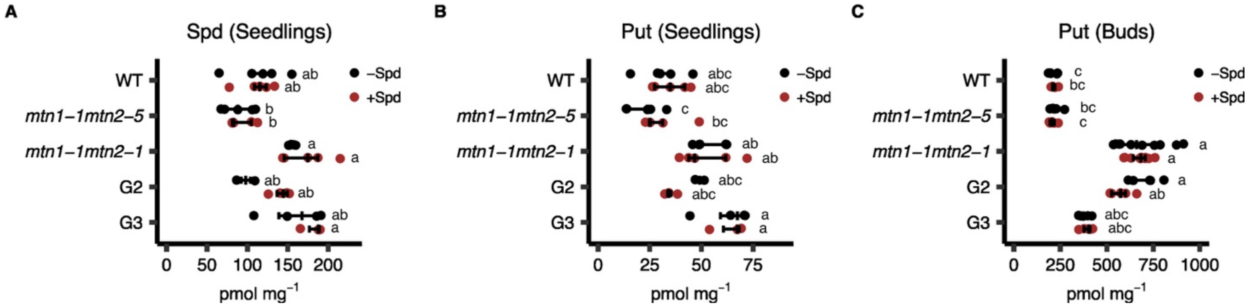

Supplementary Figure S3: Polyamine levels are not reduced in MTN-deficient plants.

A-C Metabolite quantification data of Spd in seedlings, Put seedlings, and Put in buds, respectively, from Col-0 (WT), *mtn1-1mtn2-1*, *mtn1-1mtn2-5*, G2, and G3 plants grown with or without Spd supplementation (n = 4-5). Statistical testing was performed using two-way Kruskal-Wallis tests with Dunn's post-hoc test. Groups not sharing letters are significantly different ($P < 0.05$).

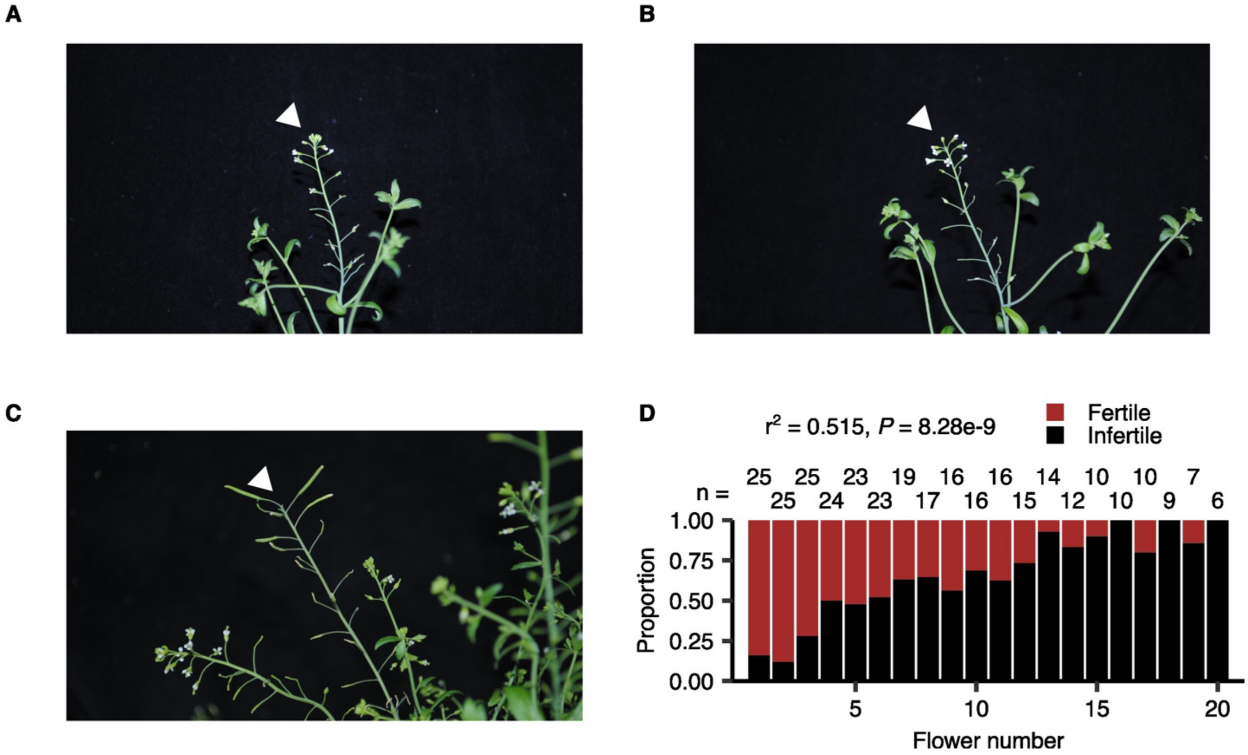

Supplementary Figure S4: Unopened bud decapitation in *mtn1-1mtn2-1* restores fertility.

A-C Photographs of a representative *mtn1-1mtn2-1* shoot before, immediately after, and 2 wk after decapitation of the apical unopened bud cluster (white arrow), respectively. **D** Relative proportion of fertile and infertile siliques on branches with a decapitated apical unopened bud cluster (n = 25). The siliques were counted in ascending order of the flowers remaining after decapitation. The data was fitted to a linear model to test whether a significant relationship exists between the proportion of fertile flowers and the distance from the site of decapitation (i.e., flower number; adjusted R-squared = 0.515, *P*-value = 8.28e-9).

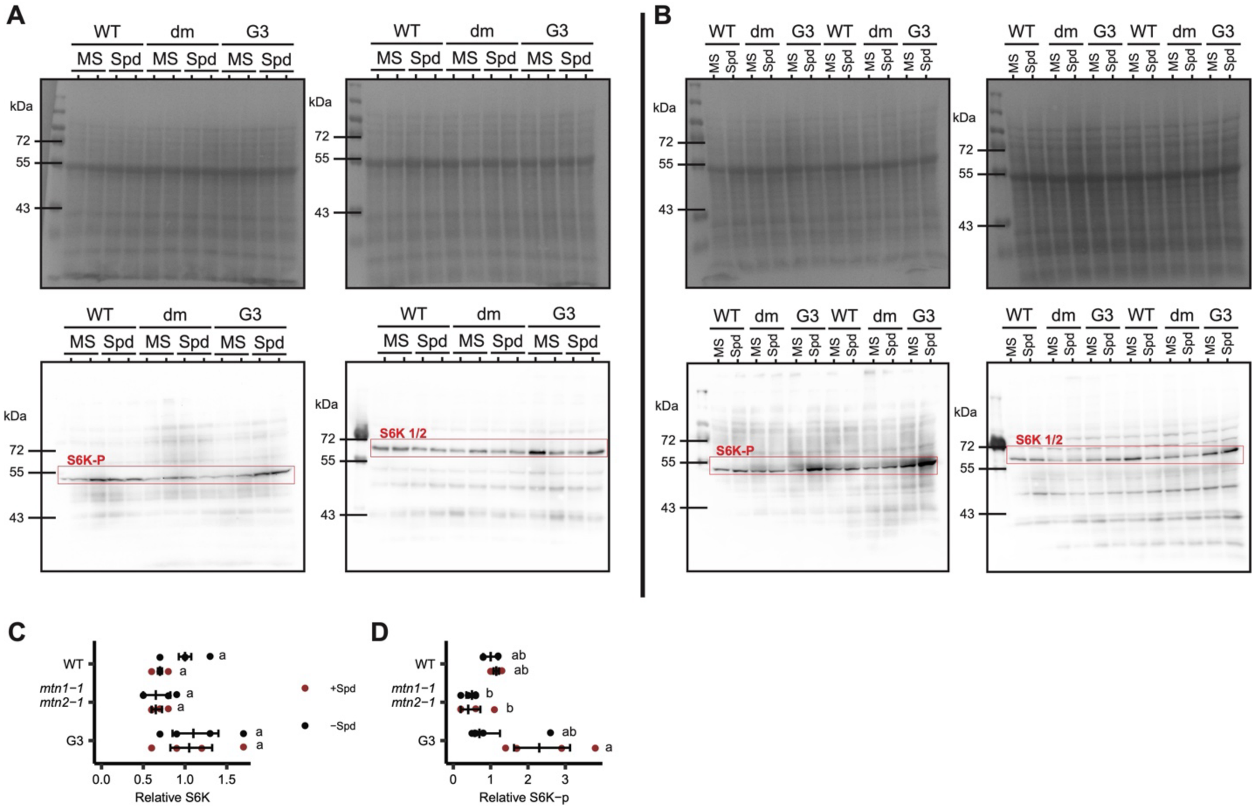

Supplementary Figure S5: S6K levels are largely not affected in *mtn1-1mtn2-1*.

A-B Raw, uncropped Coomassie Blue loading control blots and immunoblots showing unphosphorylated and phosphorylated S6K levels from Col-0 (WT), *mtn1-1mtn2-1* (dm), and G3 buds grown with or without Spd supplementation. Biological replicates 1 and 2 are shown in **A**, while replicates 3 and 4 are shown in **B**. **C-D** Relative quantification of unphosphorylated and phosphorylated S6K levels from blots shown in **A-B**, respectively, (n = 4). Statistical testing was performed using two-way Kruskal-Wallis tests with Dunn's post-hoc test. Groups not sharing letters are significantly different (P < 0.05). Protein levels are shown relative to the average levels in Col-0 buds grown without Spd supplementation.

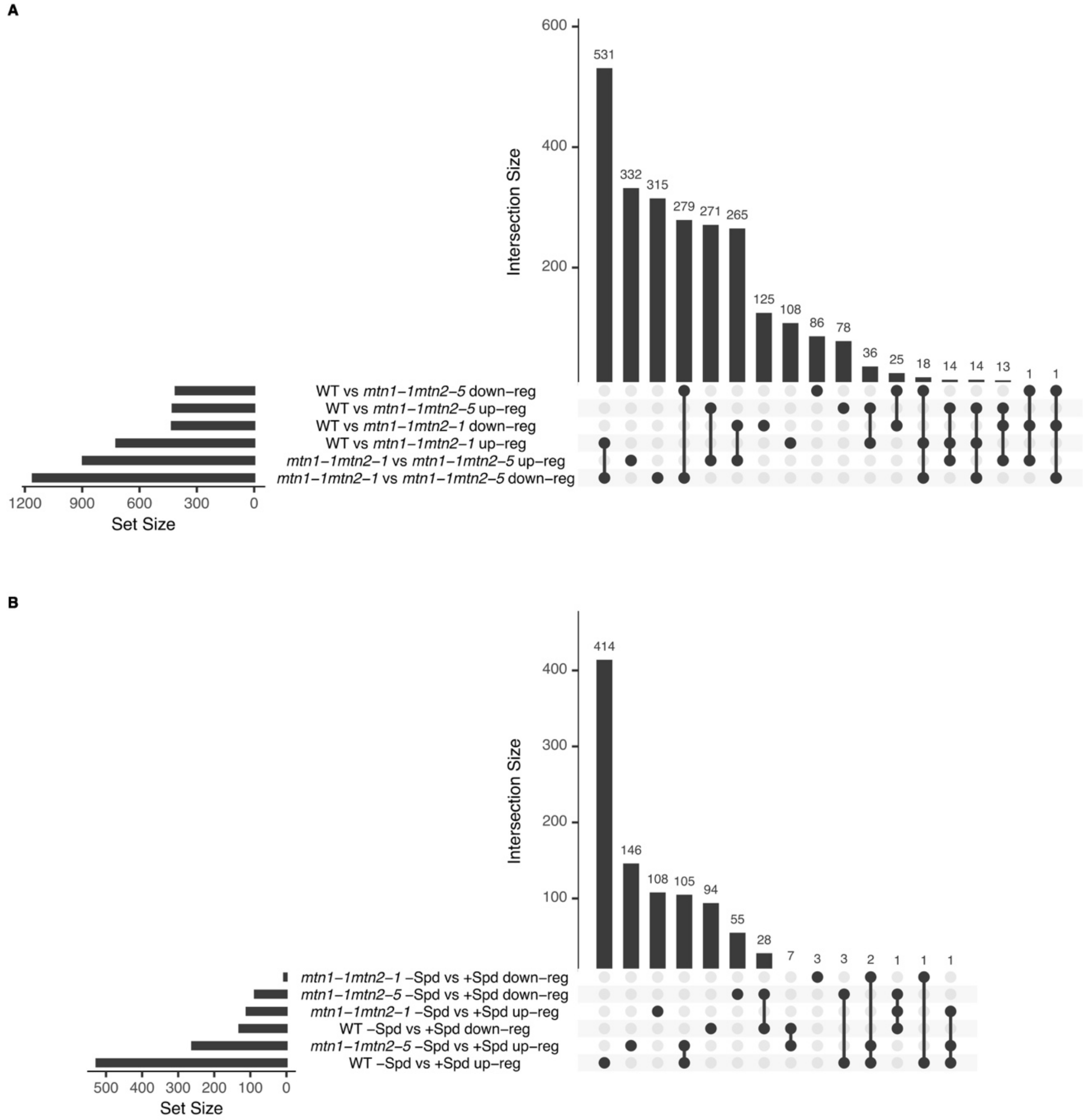

Supplementary Figure S6: Transcriptome changes in *mtn1-1mtn2-1* seedlings are minor.

A UpSet plot of overlapping gene sets in comparisons between RNA-seq of Col-0 (WT), *mtn1-1mtn2-1*, and *mtn1-1mtn2-5* seedlings without Spd supplementation. Each row refers to a list of genes from a comparison between samples, and dots in the table to the right indicate whether those genes are a part of the intersection being represented in that column. Intersections are represented as lines between dots, and those which lack connecting lines being unique to that set. **B** UpSet plot of overlapping gene sets from comparisons between treatments (with and without Spd supplementation) of samples from **A**.

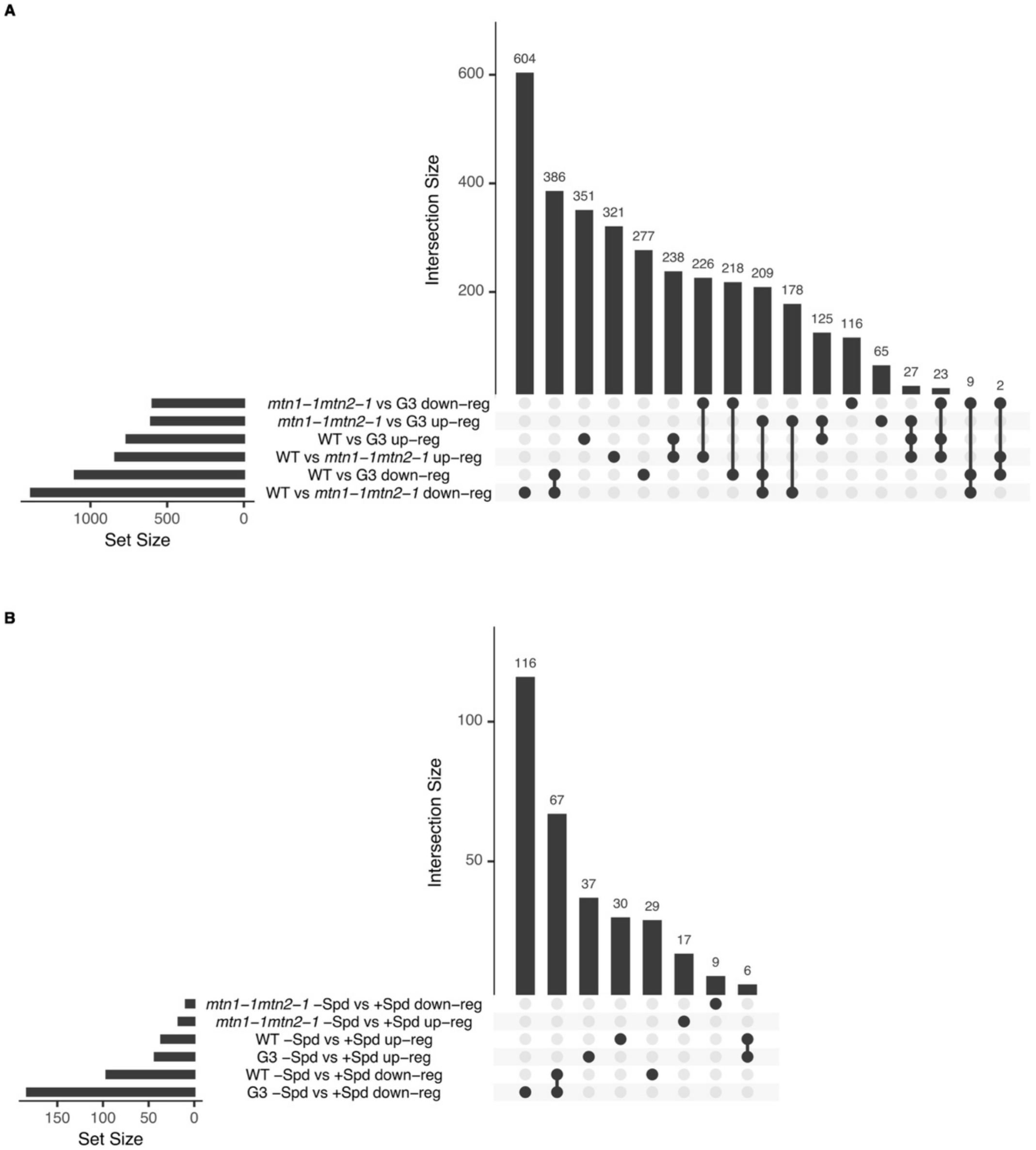

Supplementary Figure S7: The transcriptome of *mtn1-1mtn2-1* buds diverges strongly from Col-0 buds.

A UpSet plot of overlapping gene sets in comparisons between RNA-seq of Col-0 (WT), *mtn1-1mtn2-1*, and *mtn1-1mtn2-5* buds without Spd supplementation. Each row refers to a list of genes from a comparison between samples, and dots in the table to the right indicate whether those genes are a part of the intersection being represented in that column. Intersections are represented as lines between dots, and those which lack connecting lines being unique to that set. **B** UpSet plot of overlapping gene sets from comparisons between treatments (with and without Spd supplementation) of samples from **A**.

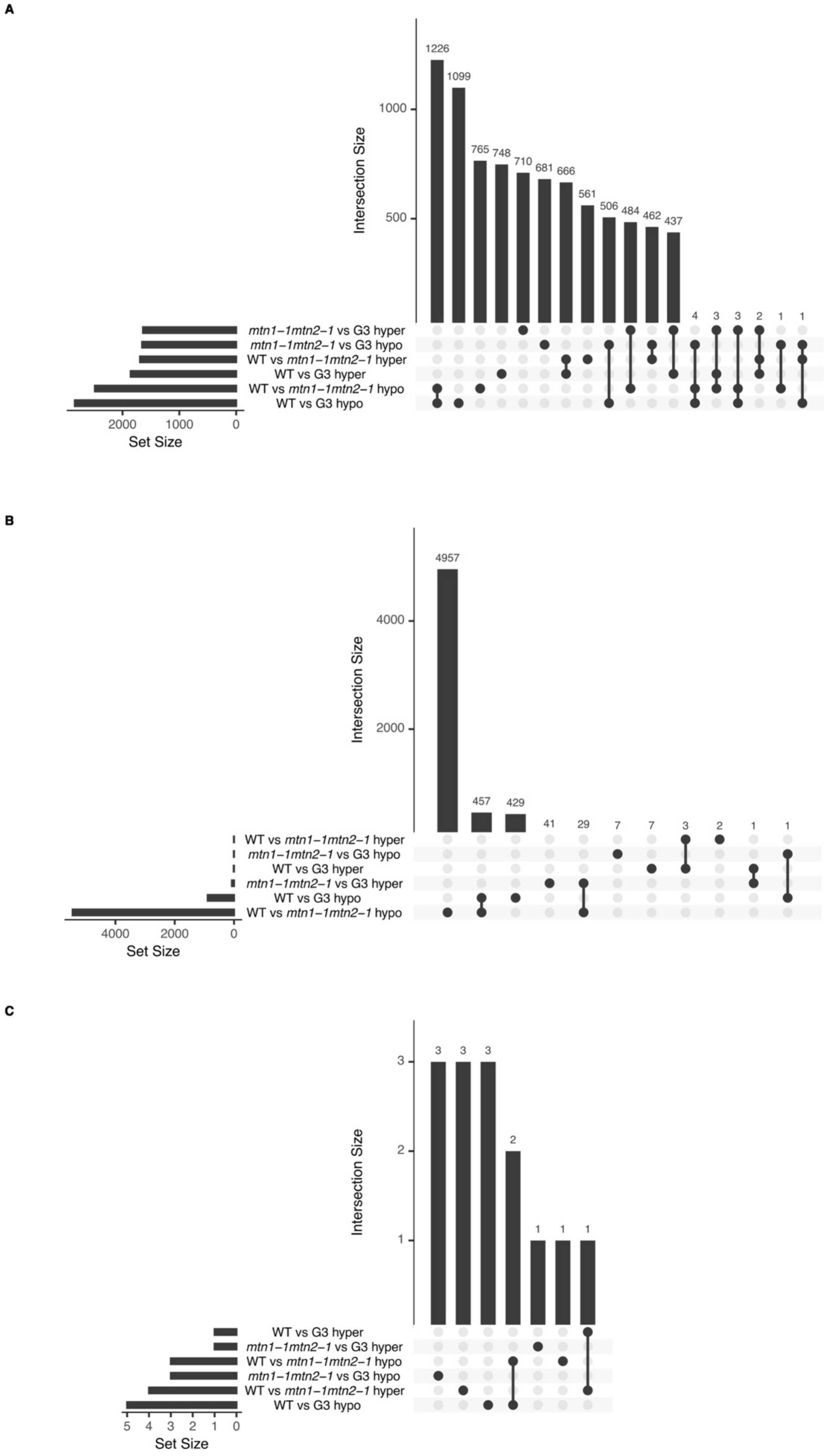
A
Intersection Size
1226
1099
765
748
710
681
666
561
506
484
462
437
4
3
3
2
1
1
mtn1−1mtn2−1 vs G3 hyper
mtn1−1mtn2−1 vs G3 hypo
WT vs mtn1−1mtn2−1 hyper
WT vs G3 hyper
WT vs mtn1−1mtn2−1 hypo
WT vs G3 hypo
Set Size
B
Intersection Size
4957
457
429
41
29
7
7
3
2
1
1
WT vs mtn1−1mtn2−1 hyper
mtn1−1mtn2−1 vs G3 hypo
WT vs G3 hyper
mtn1−1mtn2−1 vs G3 hyper
WT vs G3 hypo
WT vs mtn1−1mtn2−1 hypo
Set Size
C
Intersection Size
3
3
3
2
1
1
1
WT vs G3 hyper
mtn1−1mtn2−1 vs G3 hyper
WT vs mtn1−1mtn2−1 hypo
mtn1−1mtn2−1 vs G3 hypo
WT vs mtn1−1mtn2−1 hyper
WT vs G3 hypo
Set Size

Supplementary Figure S8: Global changes in DNA methylation in *mtn1-1mtn2-1* and G3.

A UpSet plot of overlapping sets of mCG DMRs in comparisons between BS-seq of Col-0 (WT), *mtn1-1mtn2-1*, and G3 buds without Spd supplementation. Each row refers to a list of genes from a comparison between samples, and dots in the table to the right indicate whether those genes are a part of the intersection being represented in that column. Intersections are represented as lines between dots, and those which lack connecting lines being unique to that set. **B** Same as **a** in the mCHG context. **C** Same as **A** in the mCHH context.

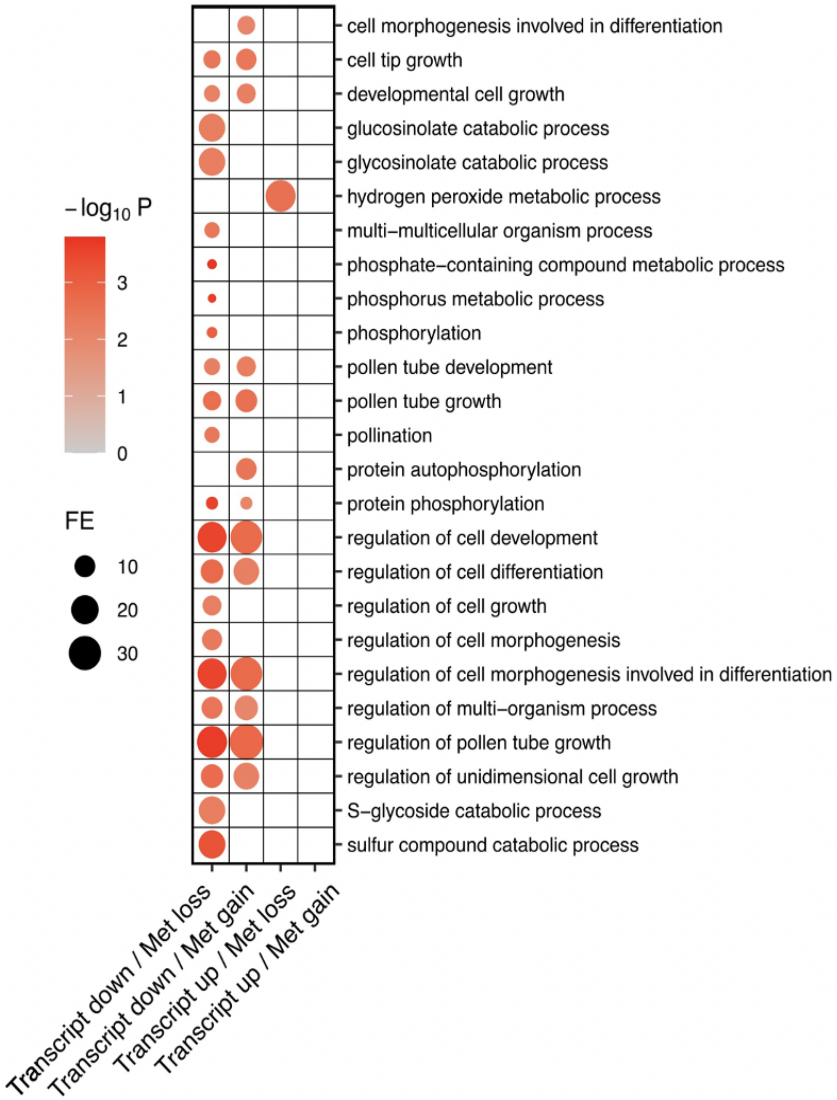

Supplementary Figure S9: Changes in DNA methylation in *mtn1-1mtn2-1* lead to changes in transcript abundance of many pollen genes.

GO enrichment of significantly differentially expressed genes in RNA-seq of *mtn1-1mtn2-1* buds with associated mCG DMRs. The colour of the circle represents the magnitude of the $-\log_{10}$ of the P-value, and the size of the circle represents the fold enrichment of genes with the associated GO term in the gene set over expected background counts.

A

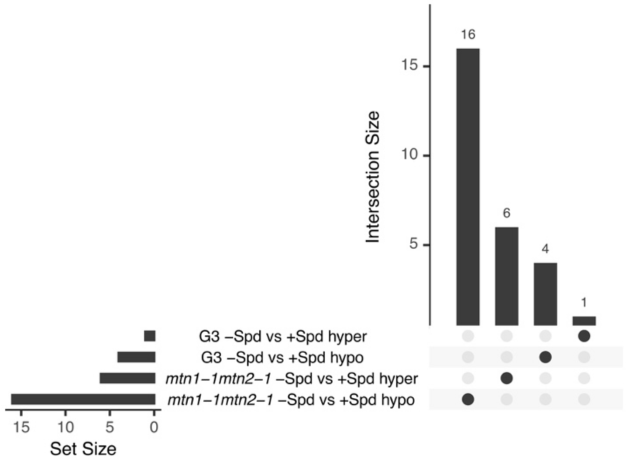

B

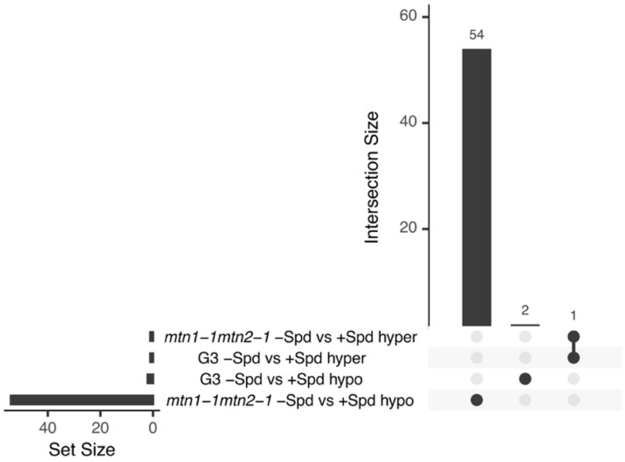

Supplementary Figure S10: The effect of within-generation Spd supplementation on DNA methylation is minor.

A UpSet plot of overlapping mCG DMRs in comparisons between BS-seq of Col-0 (WT), *mtn1-1mtn2-1*, and *mtn1-1mtn2-5* buds without Spd supplementation. Each row refers to a list of genes from a comparison between samples, and dots in the table to the right indicate whether those genes are a part of the intersection being represented in that column. Intersections are represented as lines between dots, and those which lack connecting lines being unique to that set. **B** UpSet plot of overlapping mCHG DMRs from comparisons between treatments (with and without Spd supplementation) of samples from **A**.

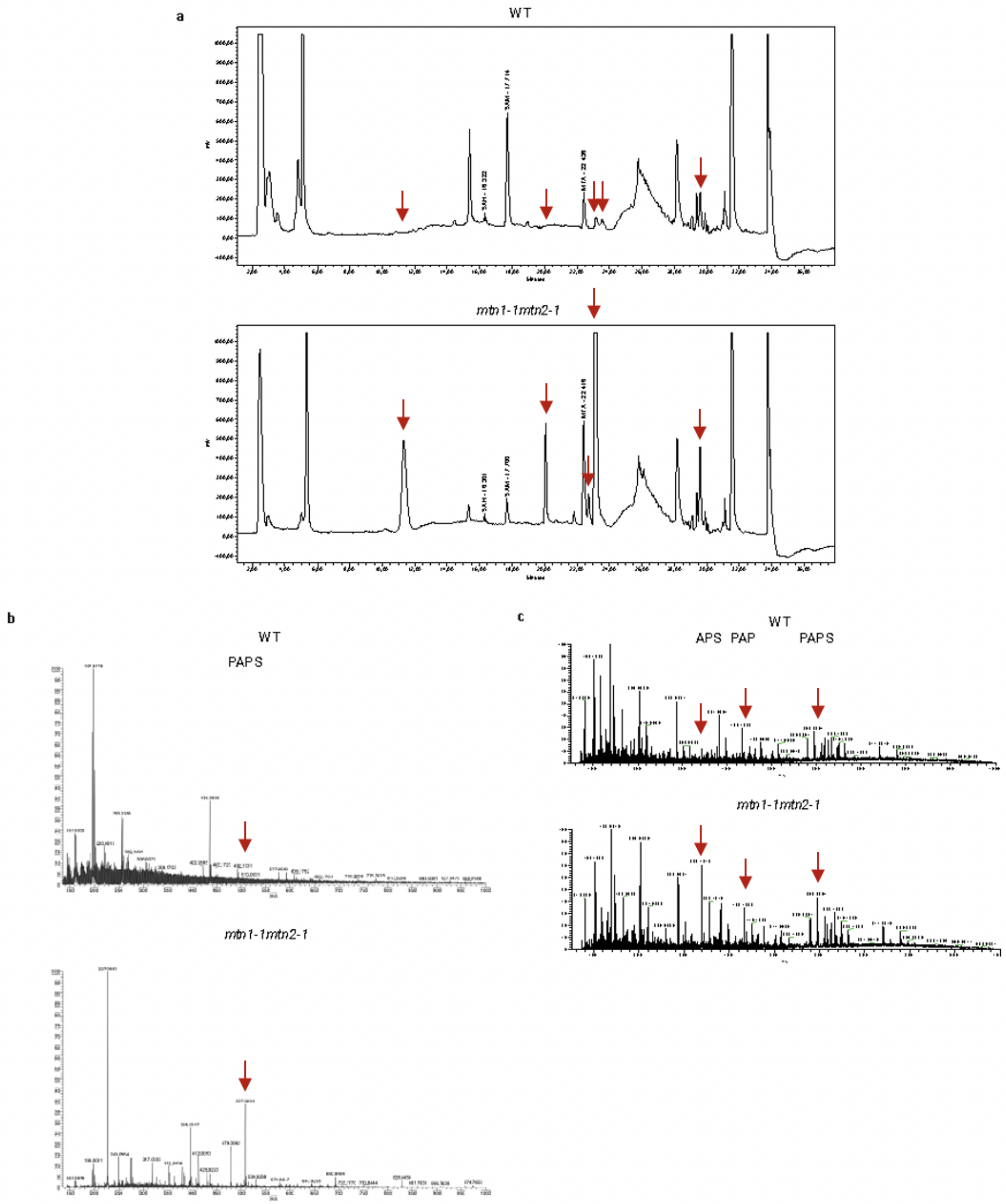

Supplementary Figure S11: Unknown peaks in *mtn1-1mtn2-1* HPLC may be indicative of increased APS and PAPS in *mtn1-1mtn2-1*.

A Representative HPLC chromatogram from WT and *mtn1-1mtn2-1* buds samples run on a Nova-Pak C18 column to quantify adenosine-containing compounds SAM, SAH, and

MTA. Arrows point to unknown peaks with increased signal in the *mtn1-1mtn2-1* samples. **B** LC/MS traces of mass (m/z) of compounds detected in WT and *mtn1-1mtn2-1* bud samples using ion trap negative mode with a Velos LTQ Orbitrap Pro (ThermoFisher Scientific). Arrows point to the putative PAPS peak with mass 507.9234 m/z. **C** Same procedure as **B** using ion trap positive mode. Arrows point to putative adenosine, APS, PAP, and PAPS peaks with mass 287.0874 m/z, 355.1719 m/z, 433.1733 m/z and 595.2594 m/z, respectively.

Supplementary Table S1: Genotyping primers used in this study.

Primer name	Description	Sequence
mtn1-1F	Genotyping SALK_085385 with LBb1.3.	TGACGGAGACCAACTCCATAC
mtn1-1R		GAGGCTCTTCCTTTGGTCAAC
mtn2-1F	Genotyping SALK_071127 with LBb1.3.	CCTTGCTTACGTGGCATAAAC
mtn2-1R		GGAAAGGGCAAAAATATATGG
mtn2-5F	Genotyping SALK_022510 with LBb1.3.	ACTGTGCCAACACTCTCAACC
mtn2-5R		AAGATTTCCGCTTCCTGAAAG
LBb1.3	Used with SALK primers.	ATTTTGCCGATTTCGGAAC

Supplementary Table S2: Correlations between MTA and other measured metabolites from buds.

**Metabolite**	**Pearson *r***	***P*-value**
Putrescine	0.93423564	9.56E-27
SAH	0.91342278	1.59E-23
Asparagine	0.69641127	1.28E-09
Glutamic acid	0.68700653	2.60E-09
Valine	0.63154314	1.06E-07
Threonine	0.61379814	3.00E-07
Proline	0.57105673	2.85E-06
Arginine	0.52480777	2.35E-05
Glutamine	0.51134743	4.10E-05
Histidine	0.50276298	5.77E-05
Glutathione	0.27313691	0.03803353
Lysine	0.23009337	0.08228297
Serine	0.22606181	0.08794777
Glycine	0.16736813	0.21334991
Phosphate	0.12932903	0.33325865
Phenylalanine	0.10076862	0.45166401
Aspartic acid	0.04863701	0.71693375
Spermidine	-0.1075407	0.42167955
Leucine	-0.1822703	0.17086981
Isoleucine	-0.2148521	0.10531345
Nitrate	-0.2165562	0.10251514
Alanine	-0.4067832	0.00153082
Sulfate	-0.4257477	8.63E-04
Cysteine	-0.5159042	3.40E-05
Tyrosine	-0.5164847	3.32E-05
SAM	-0.5506723	7.51E-06
Methionine	-0.8657232	1.79E-18
Nicotianamine	-0.9531832	3.03E-30
